# Supplementary figures and images for: Modular DNA barcoding of nanobodies enables multiplexed in situ protein imaging and high-throughput biomolecule detection
Source: eLife. 2025 Jul 22;14:RP105225. doi: 10.7554/eLife.105225 (PMC12283080; doi:10.7554/eLife.105225)

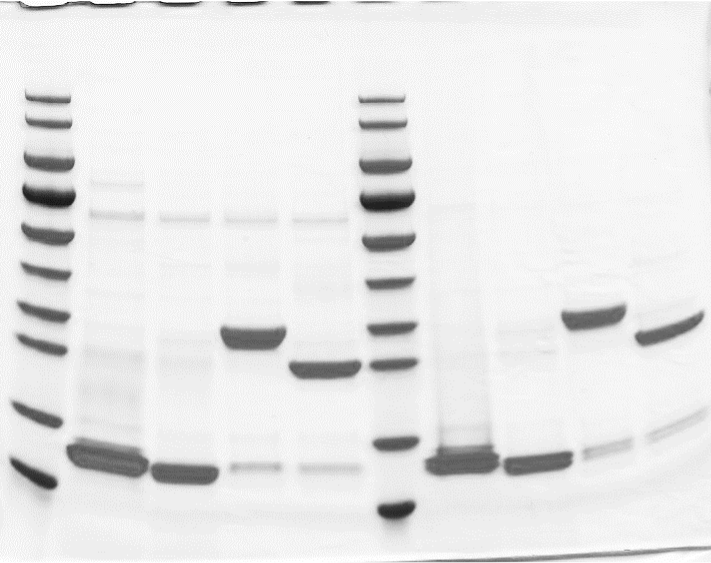

Supplement: Figure 1—source data 1. [file elife-105225-fig1-data1.zip › Figure 1_Source Data 1.png]

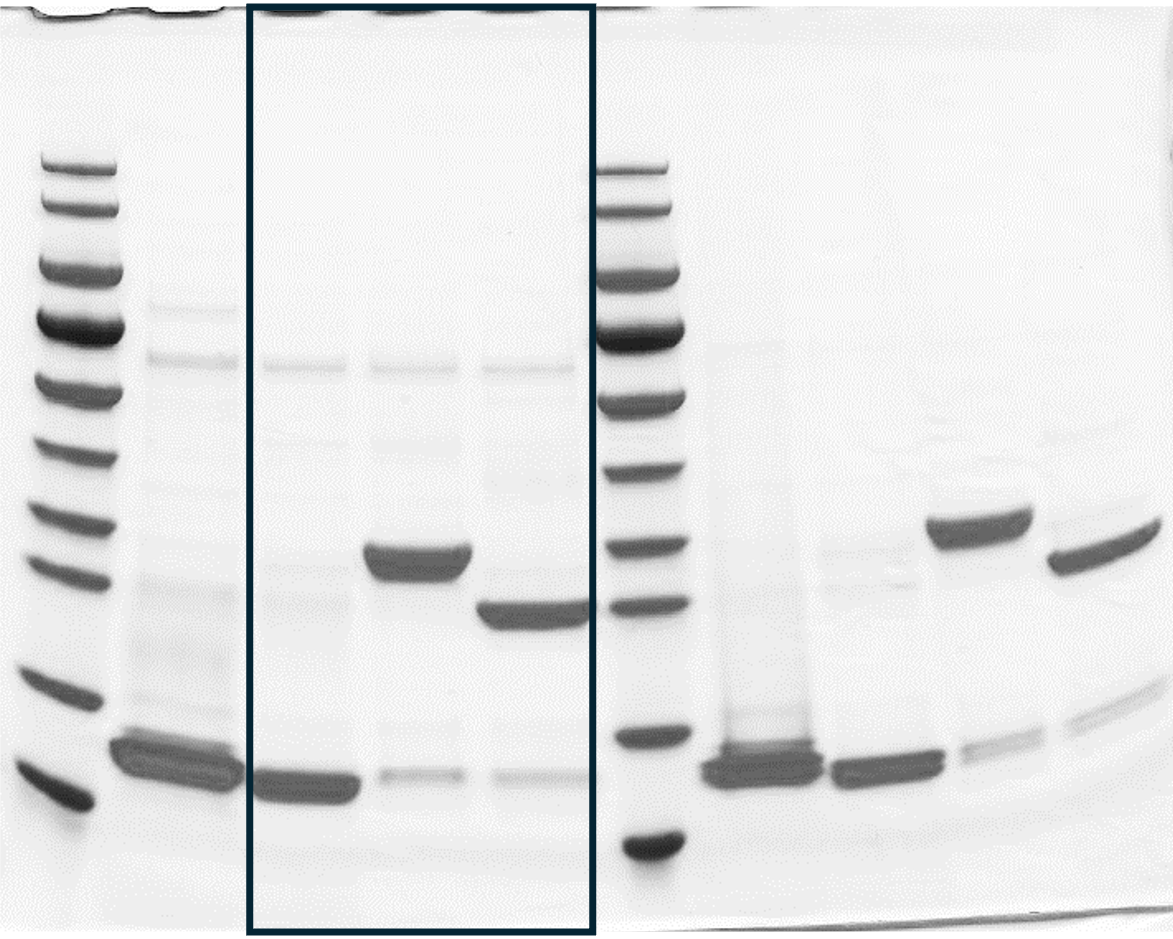

Supplement: Figure 1—source data 2. [file elife-105225-fig1-data2.zip › Figure 1_Source Data 2.png]

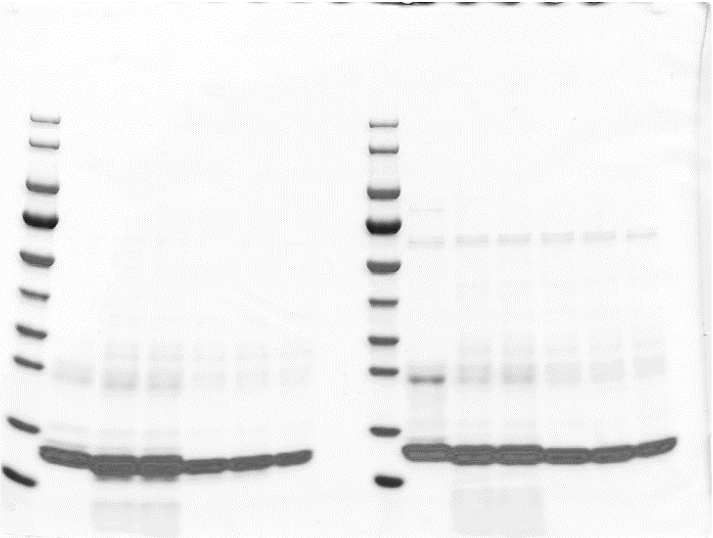

Supplement: Figure 1—figure supplement 1—source data 1. [file elife-105225-fig1-figsupp1-data1.zip › Figure 1ΓÇôFigure Supplement 1.png]

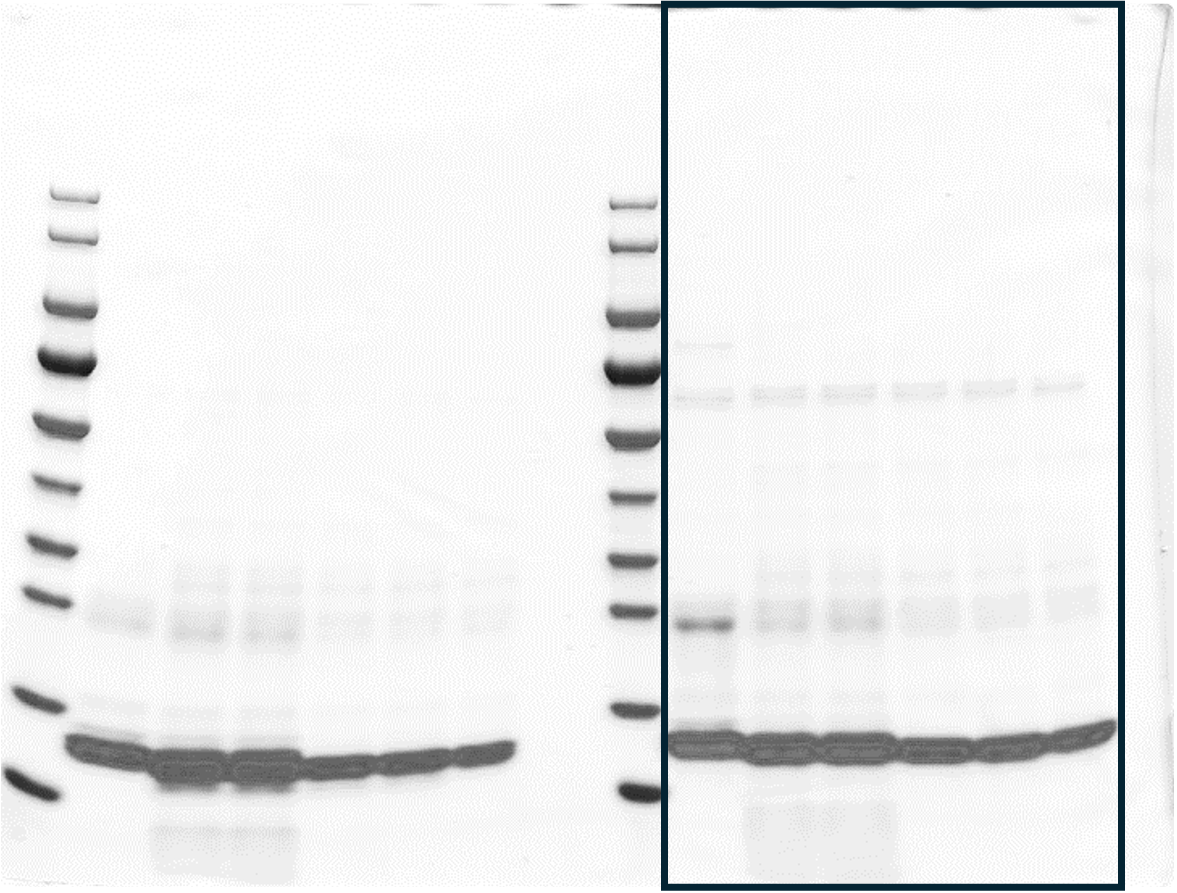

Supplement: Figure 1—figure supplement 1—source data 2. [file elife-105225-fig1-figsupp1-data2.zip › Figure 1ΓÇôFigure Supplement 2.png]

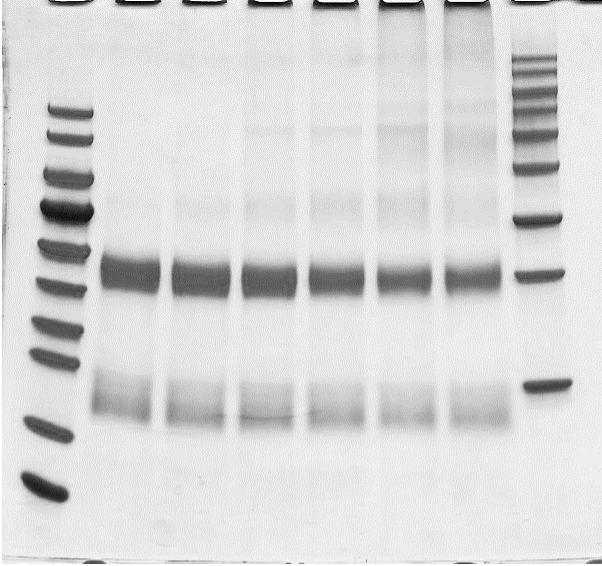

Supplement: Figure 1—figure supplement 2—source data 1. [file elife-105225-fig1-figsupp2-data1.zip › Figure 1ΓÇôFigure Supplement 2_Source Data 1_2.png]

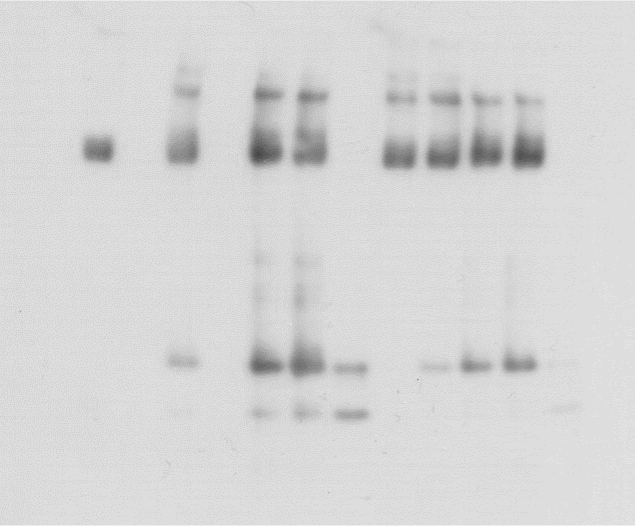

Supplement: Figure 1—figure supplement 2—source data 1. [file elife-105225-fig1-figsupp2-data1.zip › Figure 1ΓÇôFigure Supplement 2_Source Data 1_3.png]

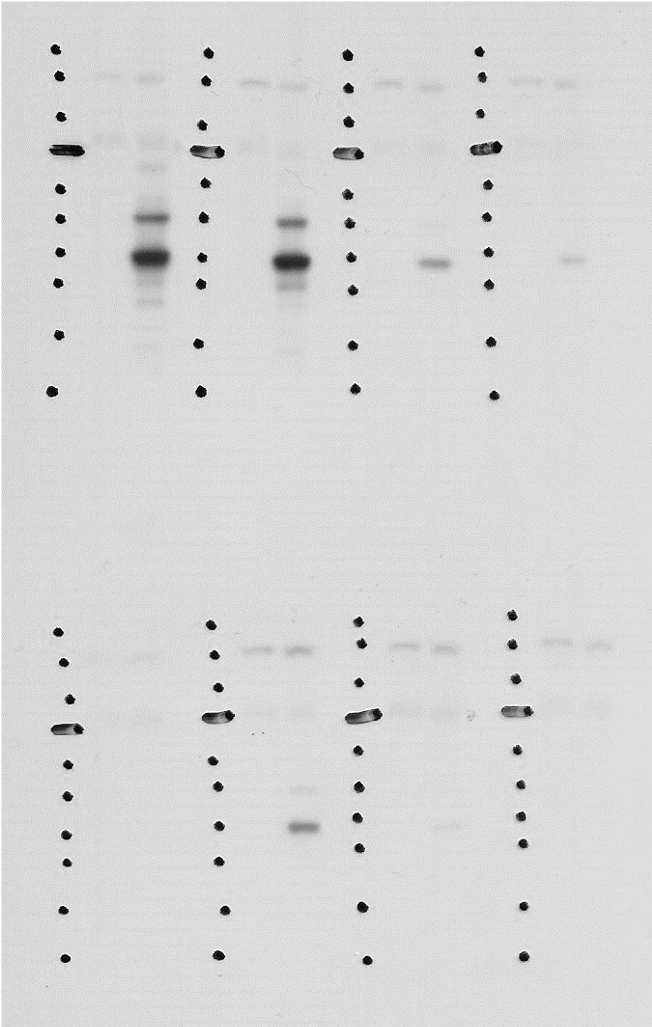

Supplement: Figure 1—figure supplement 2—source data 1. [file elife-105225-fig1-figsupp2-data1.zip › Figure 1ΓÇôFigure Supplement 2_Source Data 1_4.png]

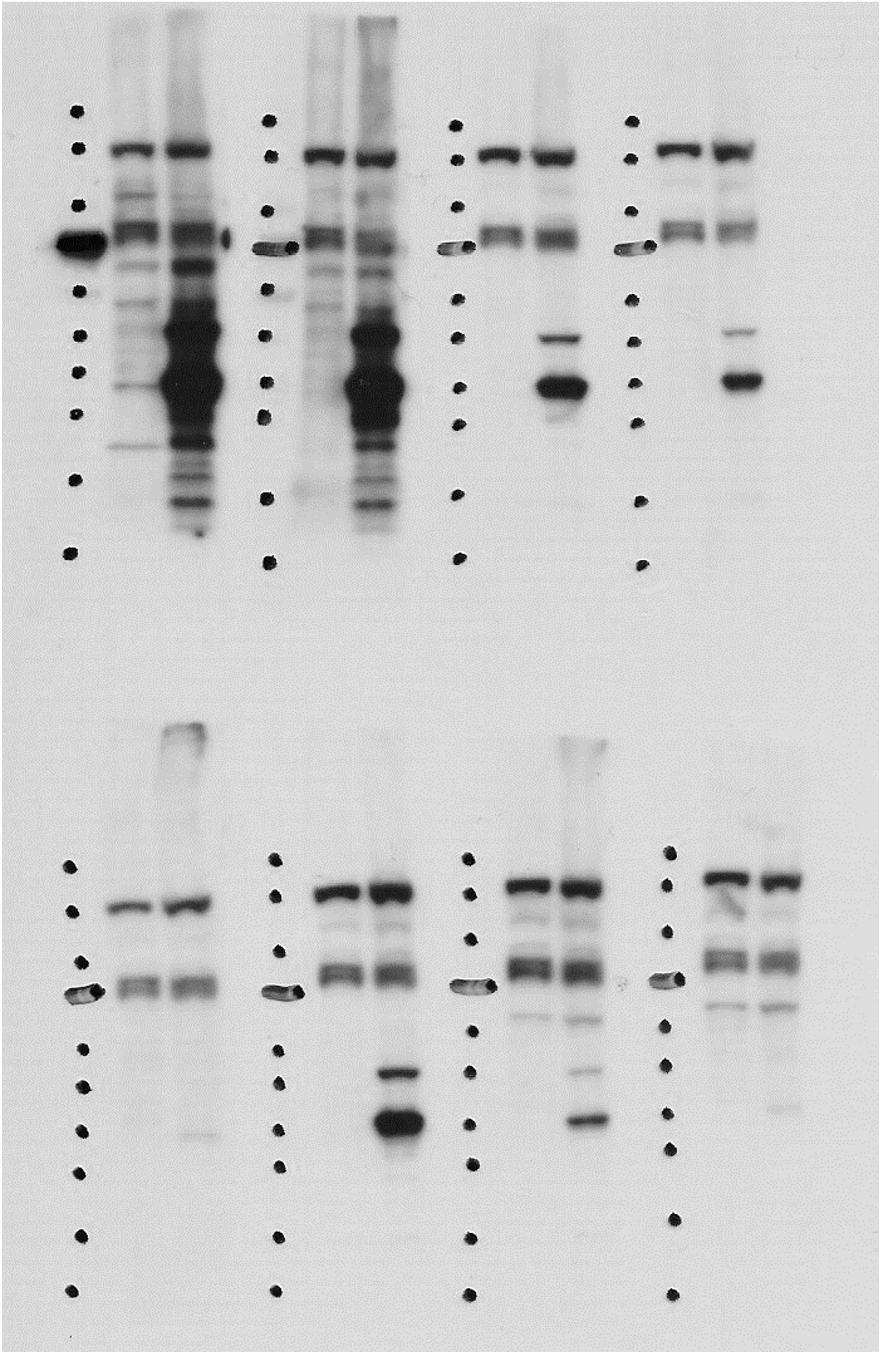

Supplement: Figure 1—figure supplement 2—source data 1. [file elife-105225-fig1-figsupp2-data1.zip › Figure 1ΓÇôFigure Supplement 2_Source Data 1_5.png]

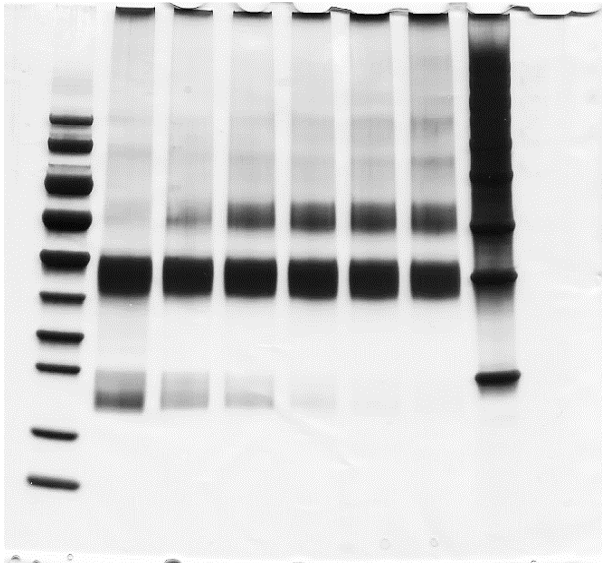

Supplement: Figure 1—figure supplement 2—source data 1. [file elife-105225-fig1-figsupp2-data1.zip › Figure 1ΓÇôFigure Supplement 2_Source Data 1.png]

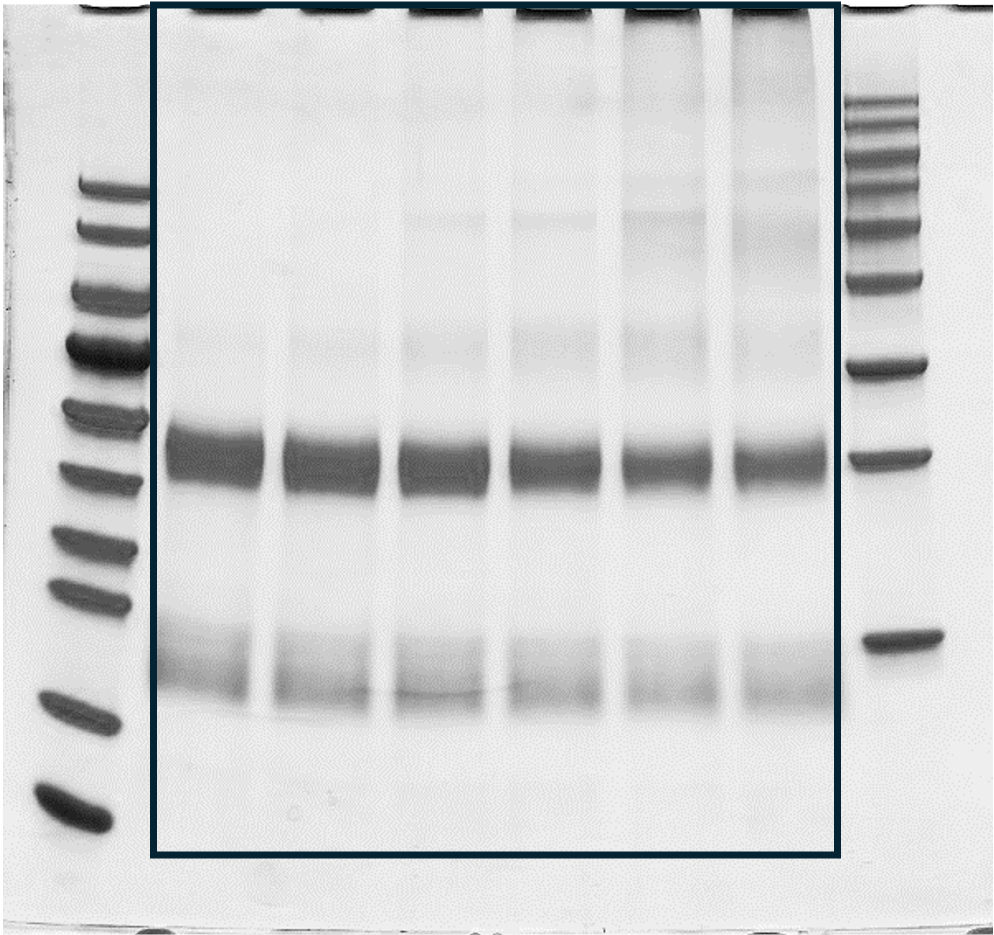

Supplement: Figure 1—figure supplement 2—source data 2. [file elife-105225-fig1-figsupp2-data2.zip › Figure 1ΓÇôFigure Supplement 2_Source Data 2_2.png]

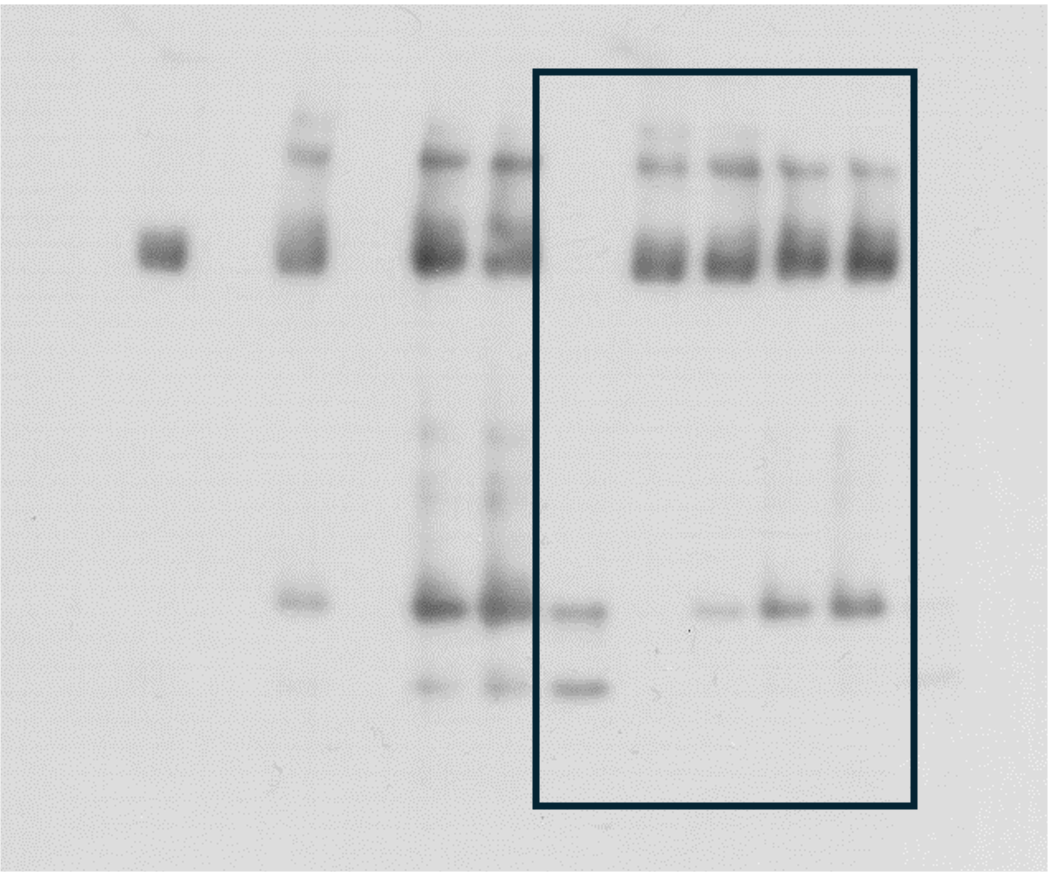

Supplement: Figure 1—figure supplement 2—source data 2. [file elife-105225-fig1-figsupp2-data2.zip › Figure 1ΓÇôFigure Supplement 2_Source Data 2_3.png]

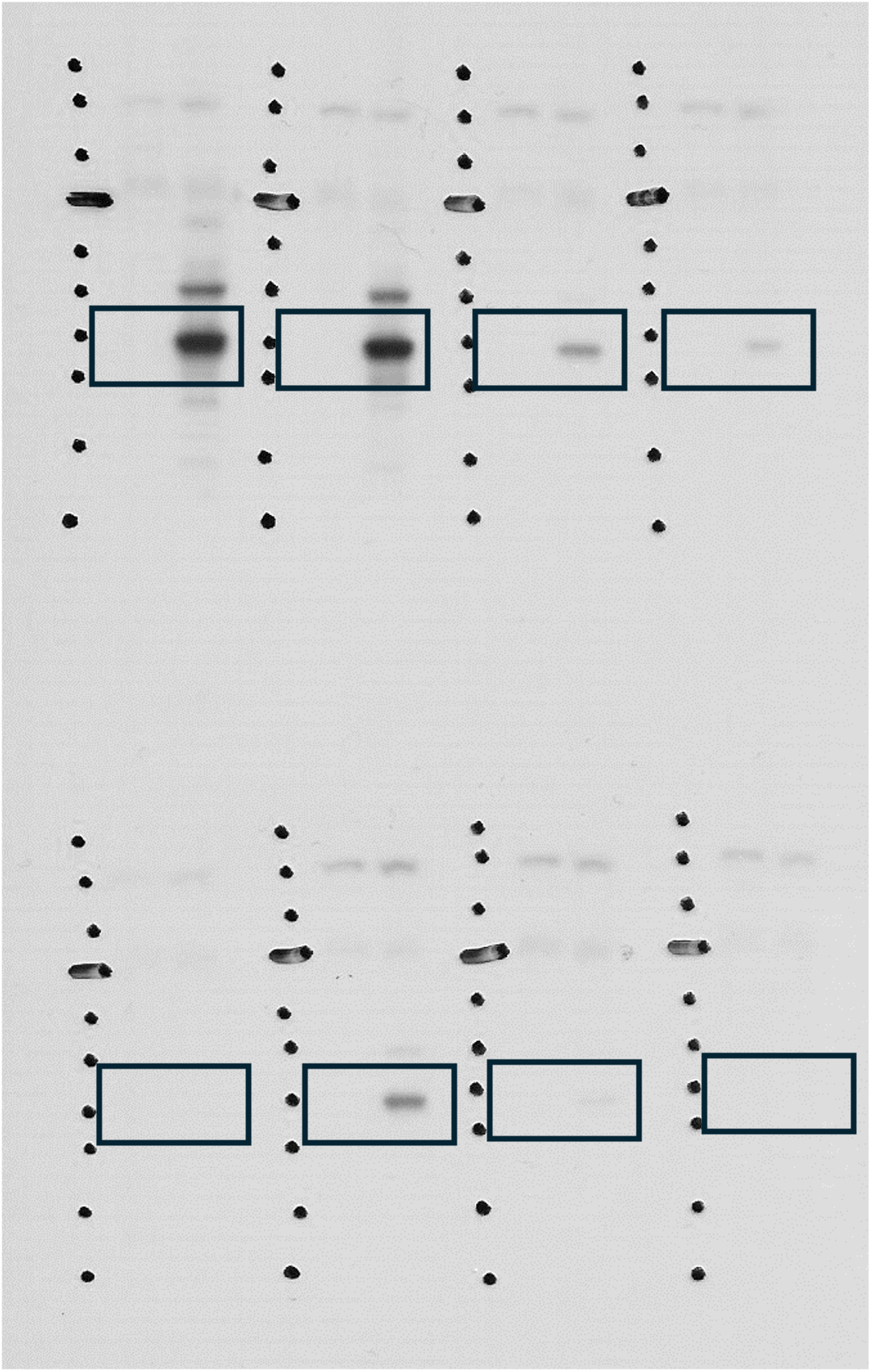

Supplement: Figure 1—figure supplement 2—source data 2. [file elife-105225-fig1-figsupp2-data2.zip › Figure 1ΓÇôFigure Supplement 2_Source Data 2_4.png]

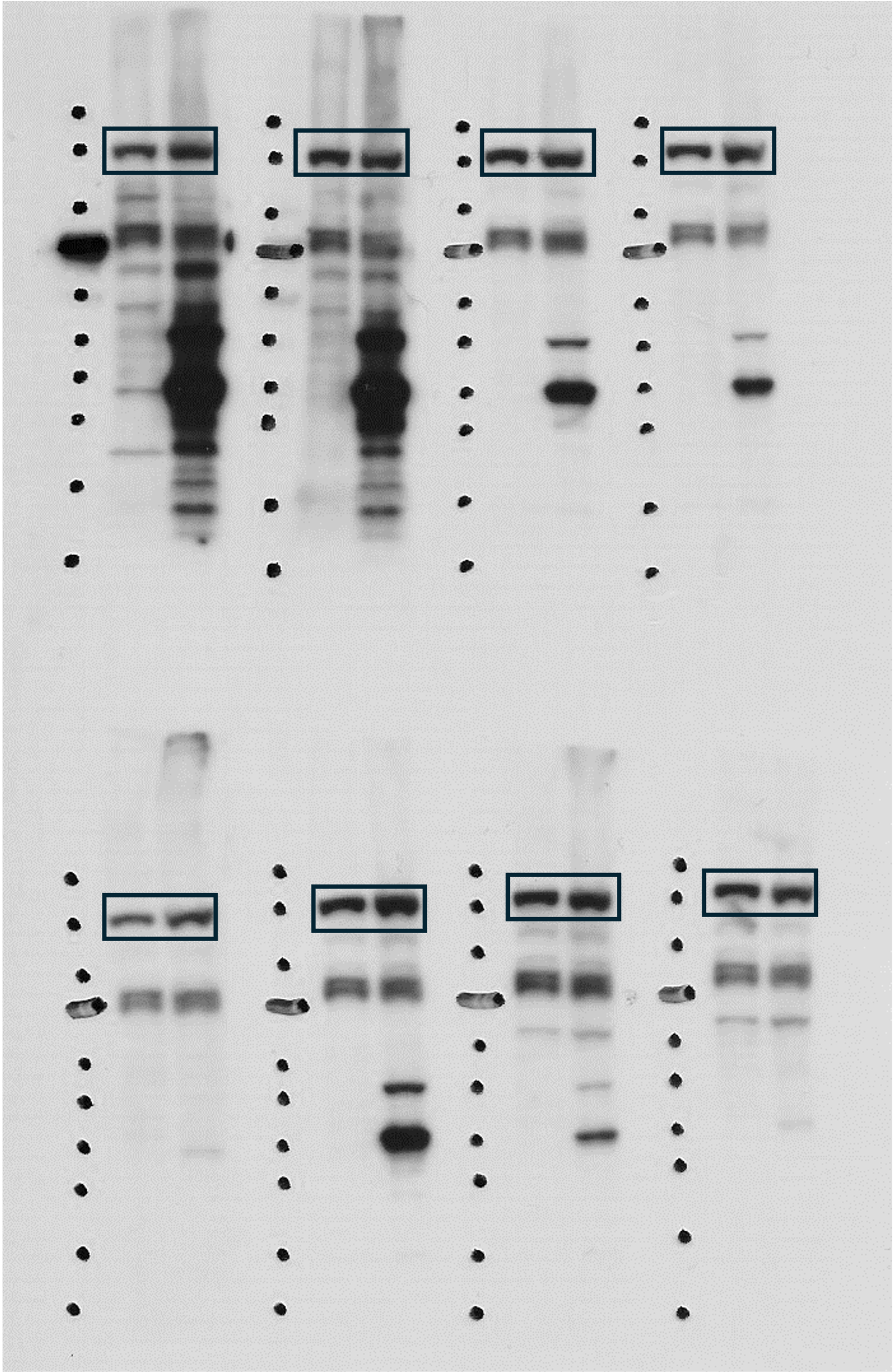

Supplement: Figure 1—figure supplement 2—source data 2. [file elife-105225-fig1-figsupp2-data2.zip › Figure 1ΓÇôFigure Supplement 2_Source Data 2_5.png]

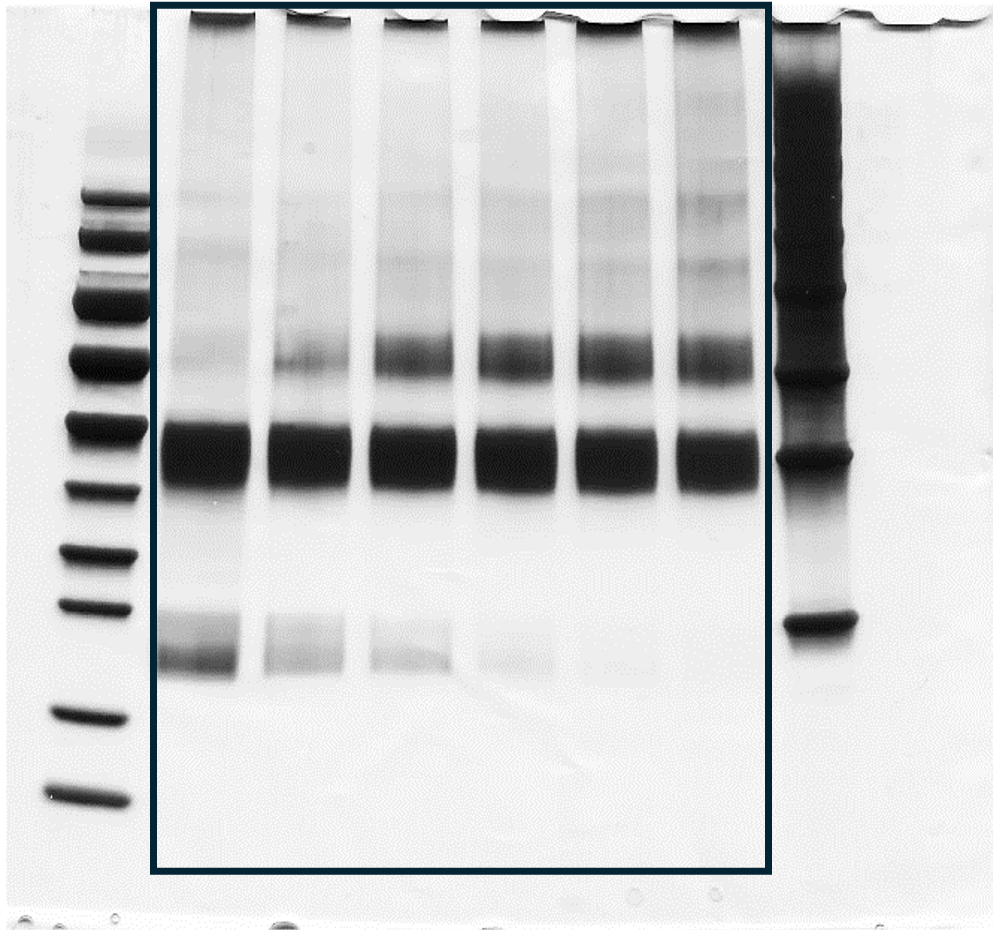

Supplement: Figure 1—figure supplement 2—source data 2. [file elife-105225-fig1-figsupp2-data2.zip › Figure 1ΓÇôFigure Supplement 2_Source Data 2.png]

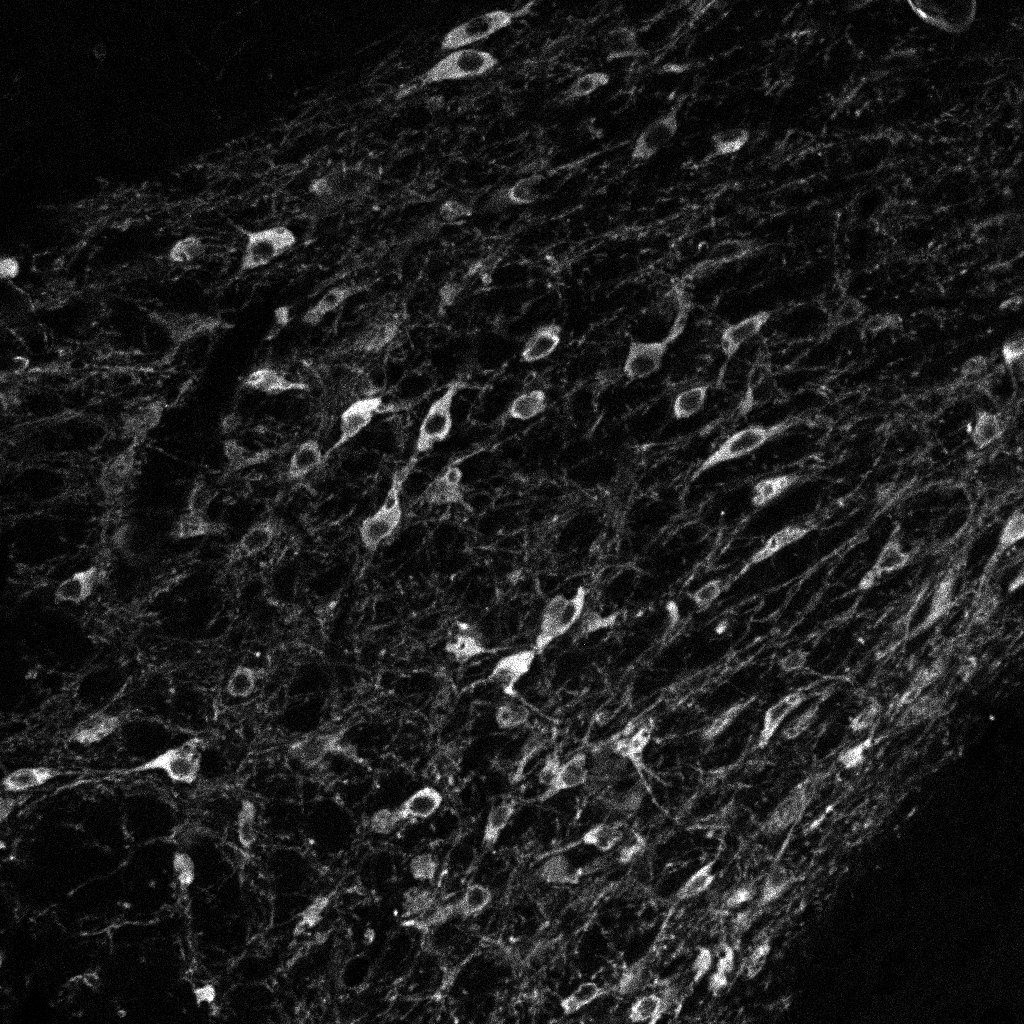

Supplement: Figure 1—figure supplement 3—source data 1. [file elife-105225-fig1-figsupp3-data1.zip › Figure 1ΓÇôFigure Supplement 3_Source Data 1/Figure 1ΓÇôFigure Supplement 3_Source Data 1_TH-Cy3 20x_higher contrast.jpg]

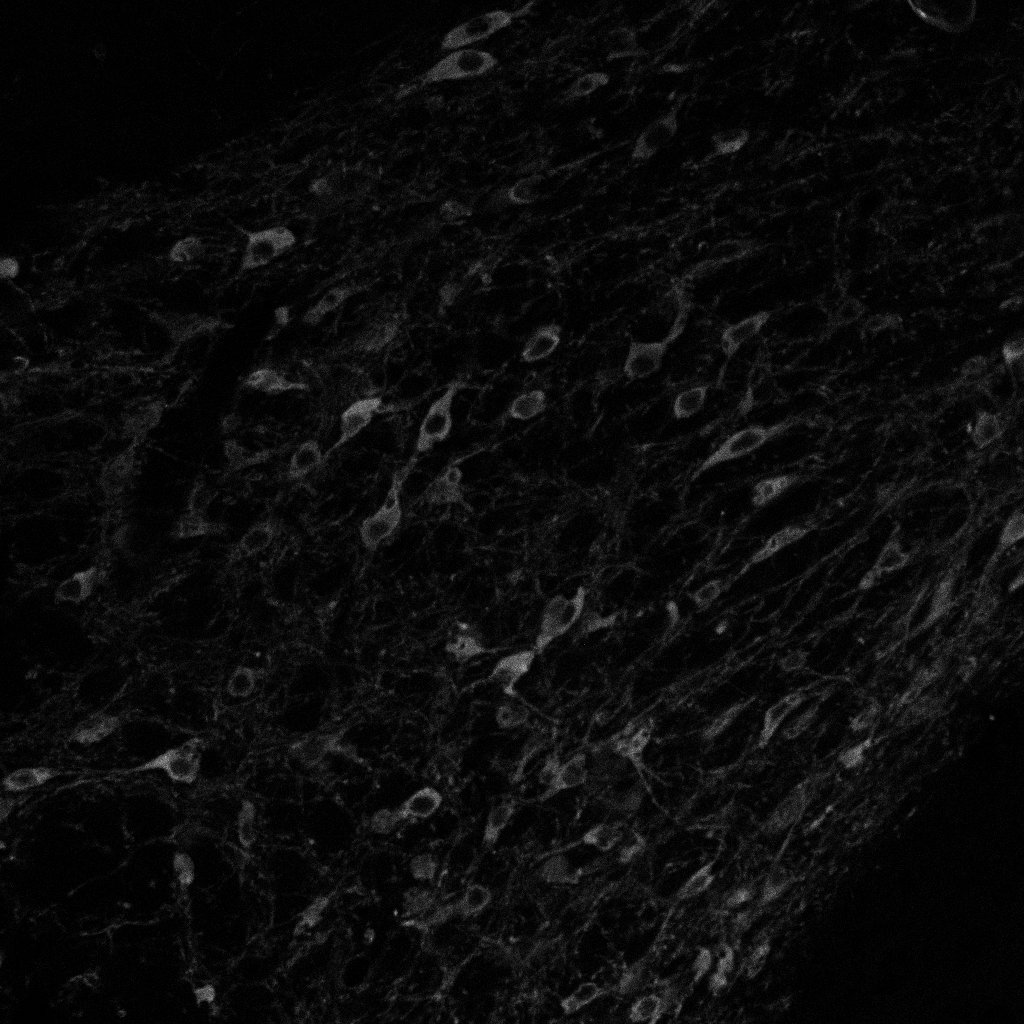

Supplement: Figure 1—figure supplement 3—source data 1. [file elife-105225-fig1-figsupp3-data1.zip › Figure 1ΓÇôFigure Supplement 3_Source Data 1/Figure 1ΓÇôFigure Supplement 3_Source Data 1_TH-Cy3 20x.jpg]

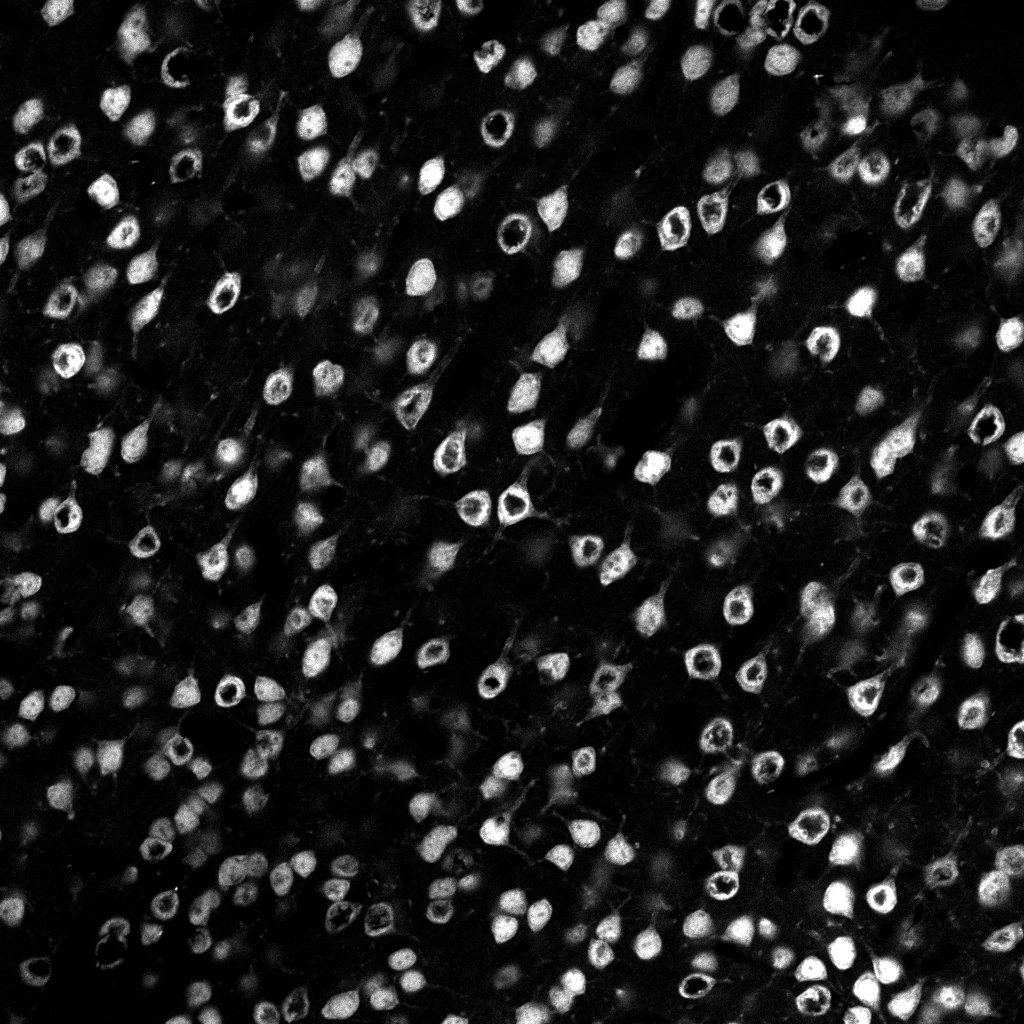

Supplement: Figure 1—figure supplement 3—source data 1. [file elife-105225-fig1-figsupp3-data1.zip › Figure 1ΓÇôFigure Supplement 3_Source Data 1/Figure 1ΓÇôFigure Supplement 3_Source Data 1_NeuN 897-B4-Cy5_20x-3.jpg]

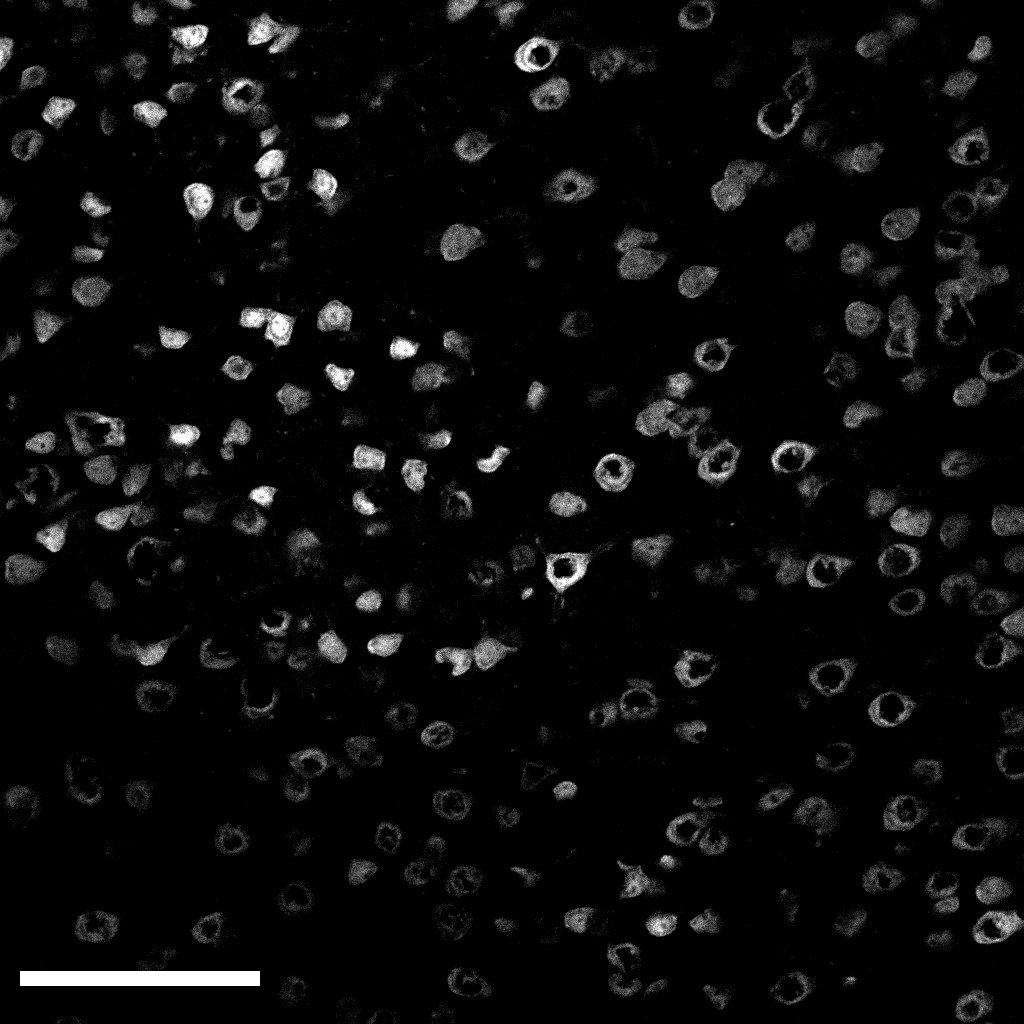

Supplement: Figure 1—figure supplement 3—source data 1. [file elife-105225-fig1-figsupp3-data1.zip › Figure 1ΓÇôFigure Supplement 3_Source Data 1/Figure 1ΓÇôFigure Supplement 3_Source Data 1_NeuN 897-Cy5_20x-1_higher contrast_scale bar 100um.jpg]

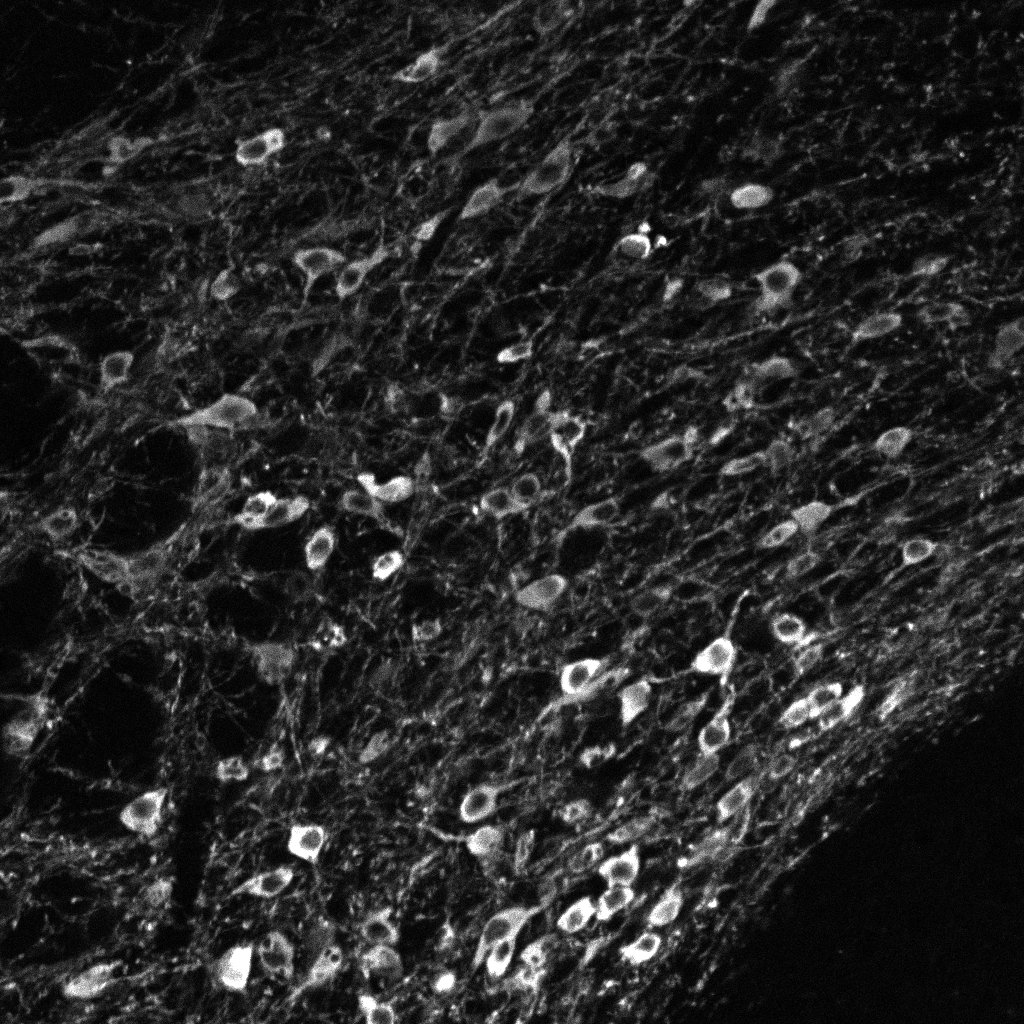

Supplement: Figure 1—figure supplement 3—source data 1. [file elife-105225-fig1-figsupp3-data1.zip › Figure 1ΓÇôFigure Supplement 3_Source Data 1/Figure 1ΓÇôFigure Supplement 3_Source Data 1_TH-B1-Cy3 20x.jpg]

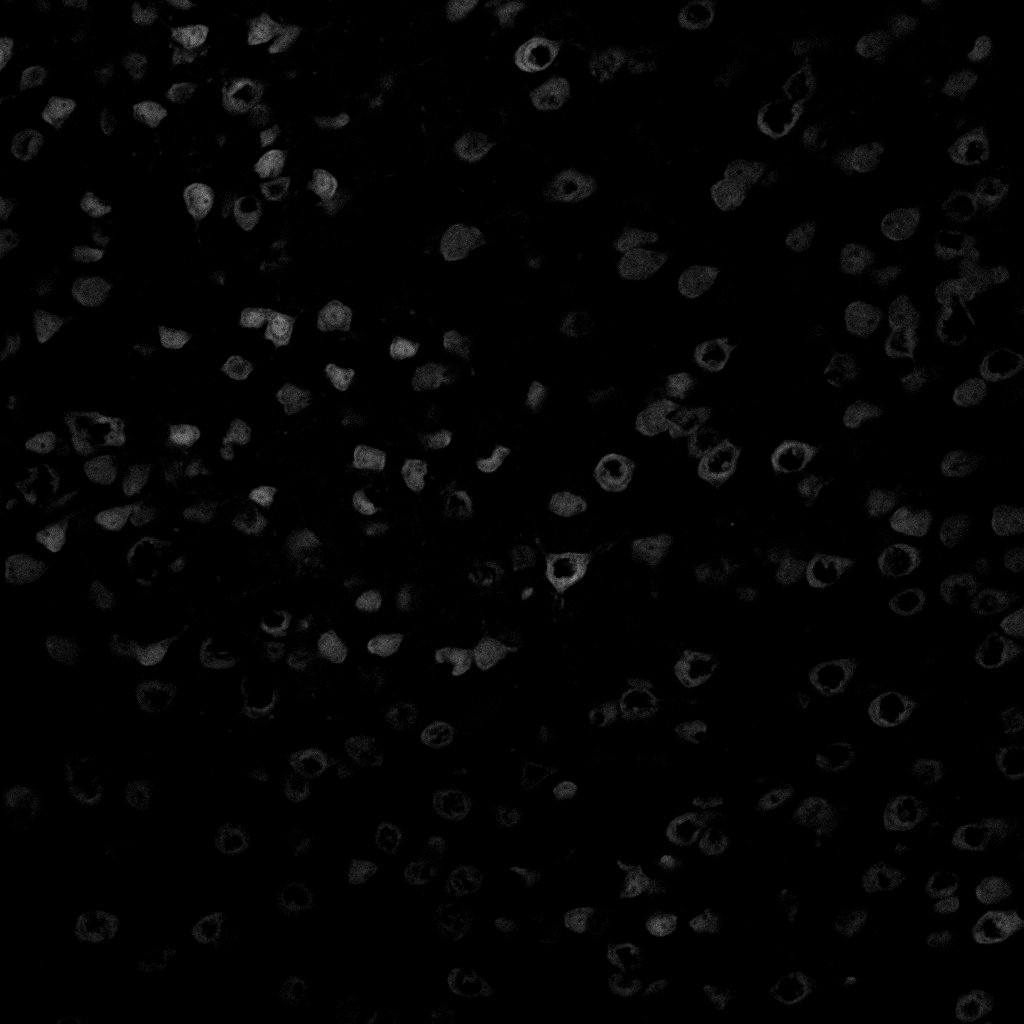

Supplement: Figure 1—figure supplement 3—source data 1. [file elife-105225-fig1-figsupp3-data1.zip › Figure 1ΓÇôFigure Supplement 3_Source Data 1/Figure 1ΓÇôFigure Supplement 3_Source Data 1_NeuN 897-Cy5_20x-1.jpg]

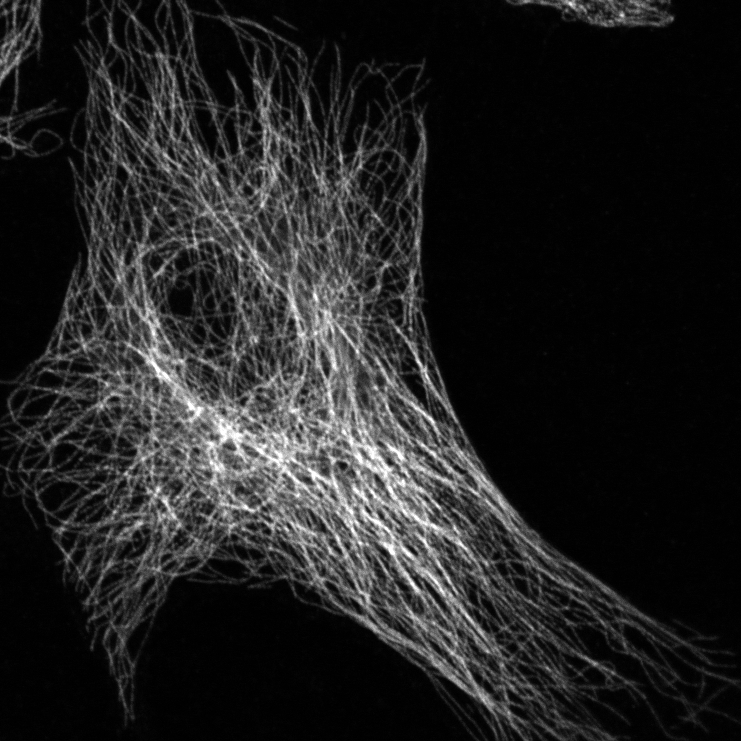

Supplement: Figure 1—figure supplement 3—source data 2. [file elife-105225-fig1-figsupp3-data2.zip › Figure 1ΓÇôFigure Supplement 3_Source Data 2/Figure 1ΓÇôFigure Supplement 3_Source Data 2_misHCR_tubulin_B4_647_100x-2_scale bar10um.tif]

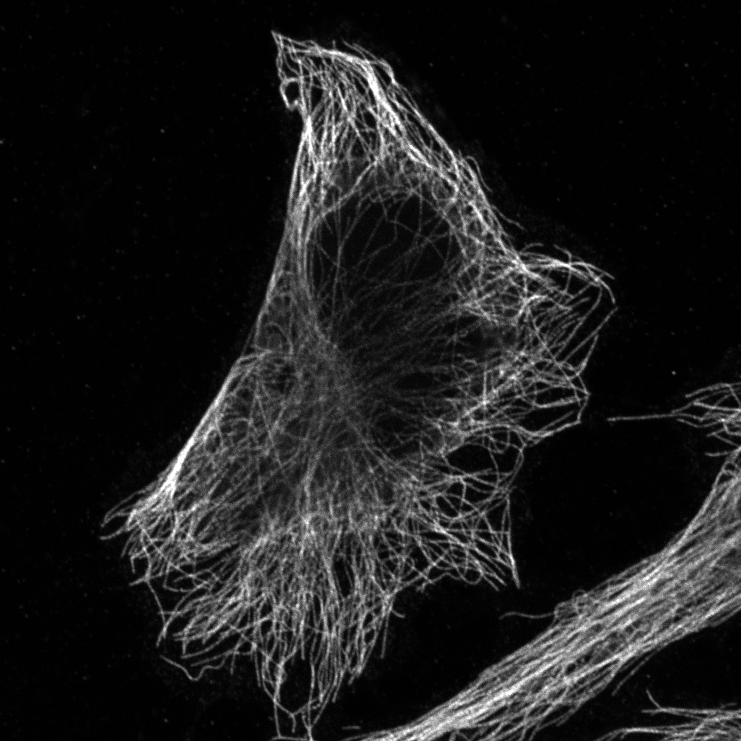

Supplement: Figure 1—figure supplement 3—source data 2. [file elife-105225-fig1-figsupp3-data2.zip › Figure 1ΓÇôFigure Supplement 3_Source Data 2/Figure 1ΓÇôFigure Supplement 3_Source Data 2_2ndAb_tubulin_GAms-647_100x-1_scale bar10um.tif]

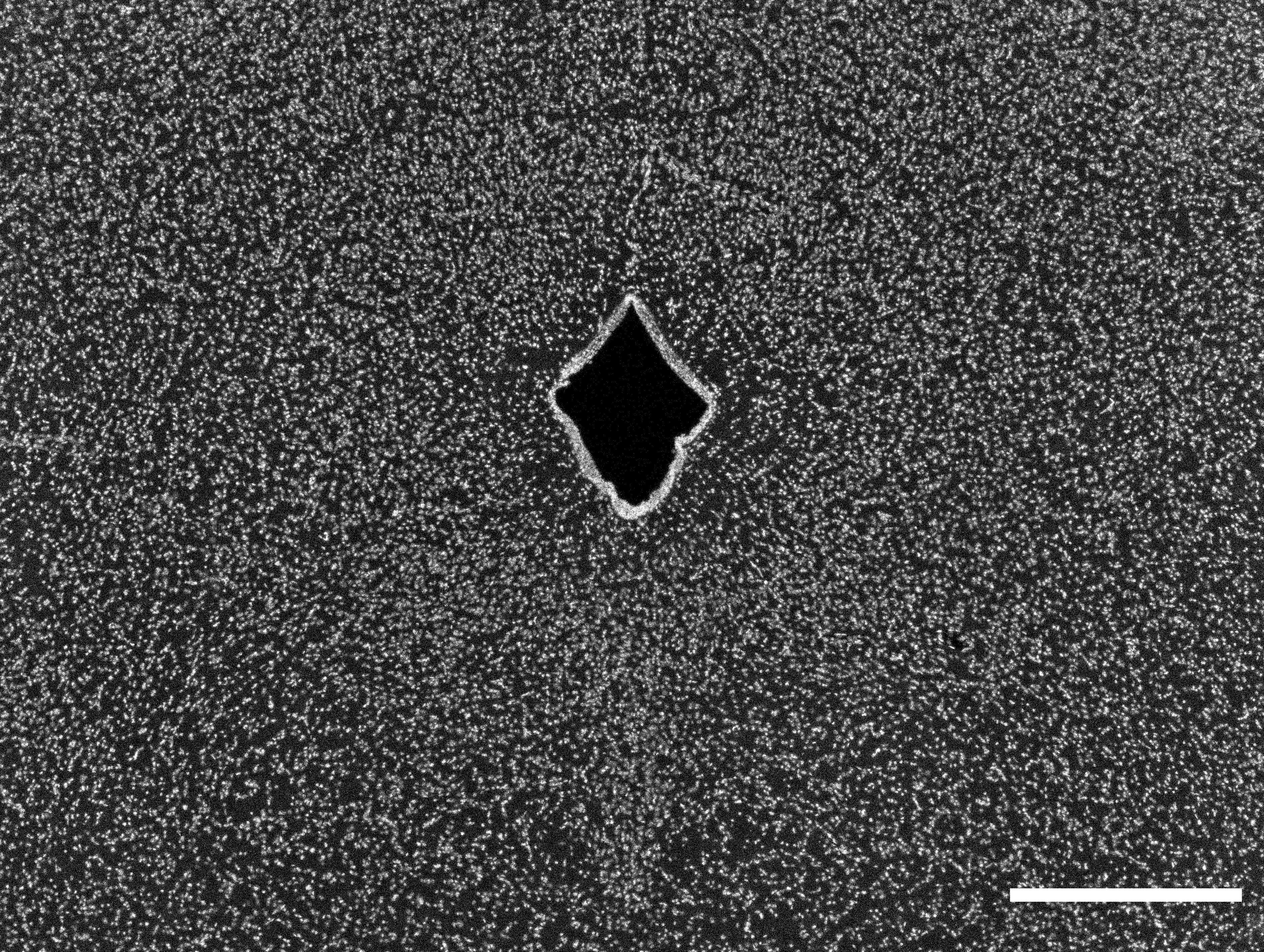

Supplement: Figure 1—figure supplement 4—source data 1. [file elife-105225-fig1-figsupp4-data1.zip › Figure 1ΓÇôFigure Supplement 4_Source Data 1/Figure 1ΓÇôFigure Supplement 4_Source Data 1_10x_DAPI NeuN TH NF-H round1_removal_DAPI.jpg]

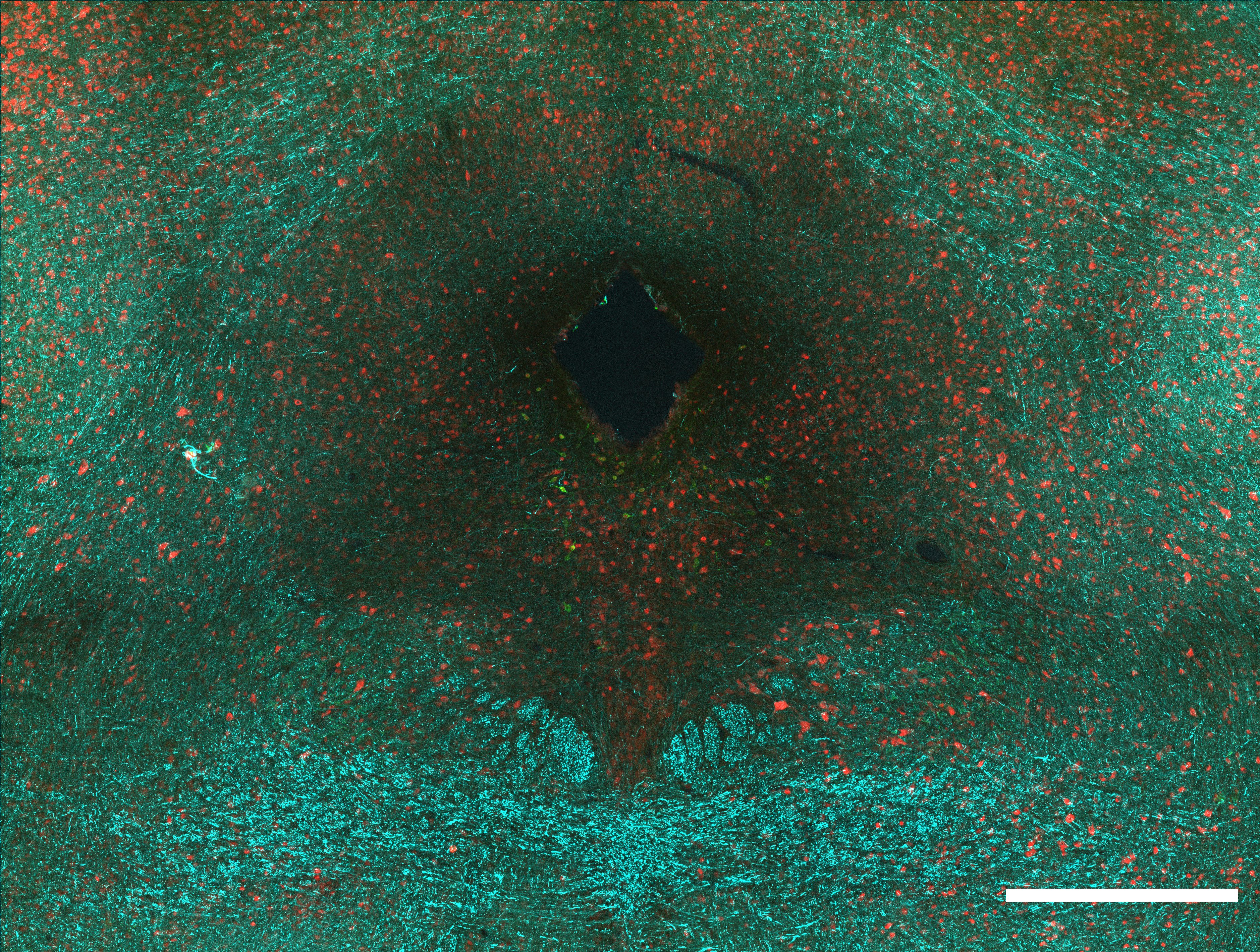

Supplement: Figure 1—figure supplement 4—source data 1. [file elife-105225-fig1-figsupp4-data1.zip › Figure 1ΓÇôFigure Supplement 4_Source Data 1/Figure 1ΓÇôFigure Supplement 4_Source Data 1_10x_DAPI NeuN TH NF-H round1_scale bar 500um.jpg]

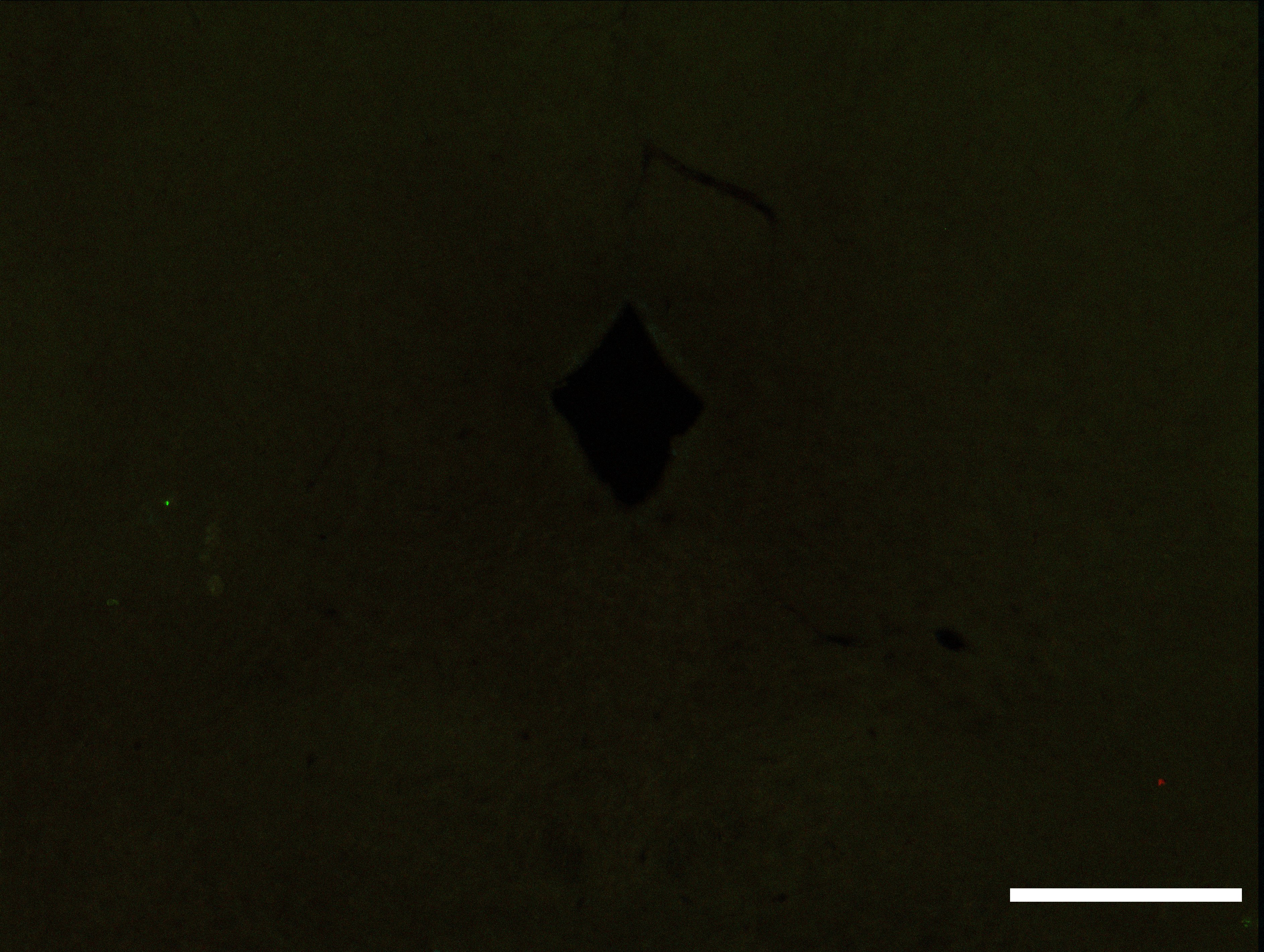

Supplement: Figure 1—figure supplement 4—source data 1. [file elife-105225-fig1-figsupp4-data1.zip › Figure 1ΓÇôFigure Supplement 4_Source Data 1/Figure 1ΓÇôFigure Supplement 4_Source Data 1_10x_DAPI NeuN TH NF-H round1_removal_scale bar 500um.jpg]

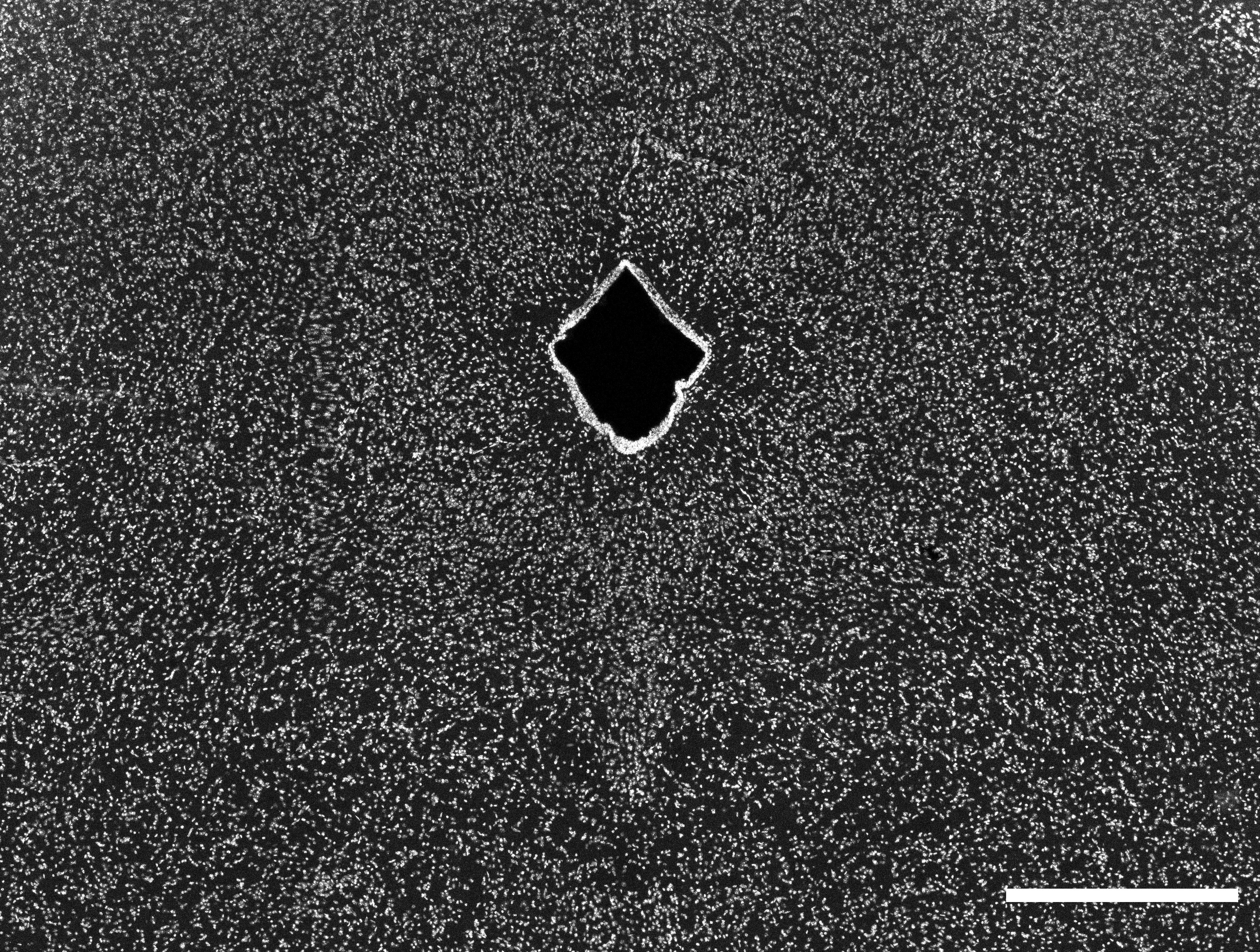

Supplement: Figure 1—figure supplement 4—source data 1. [file elife-105225-fig1-figsupp4-data1.zip › Figure 1ΓÇôFigure Supplement 4_Source Data 1/Figure 1ΓÇôFigure Supplement 4_Source Data 1_10x_DAPI NeuN TH NF-H round1_DAPI.jpg]

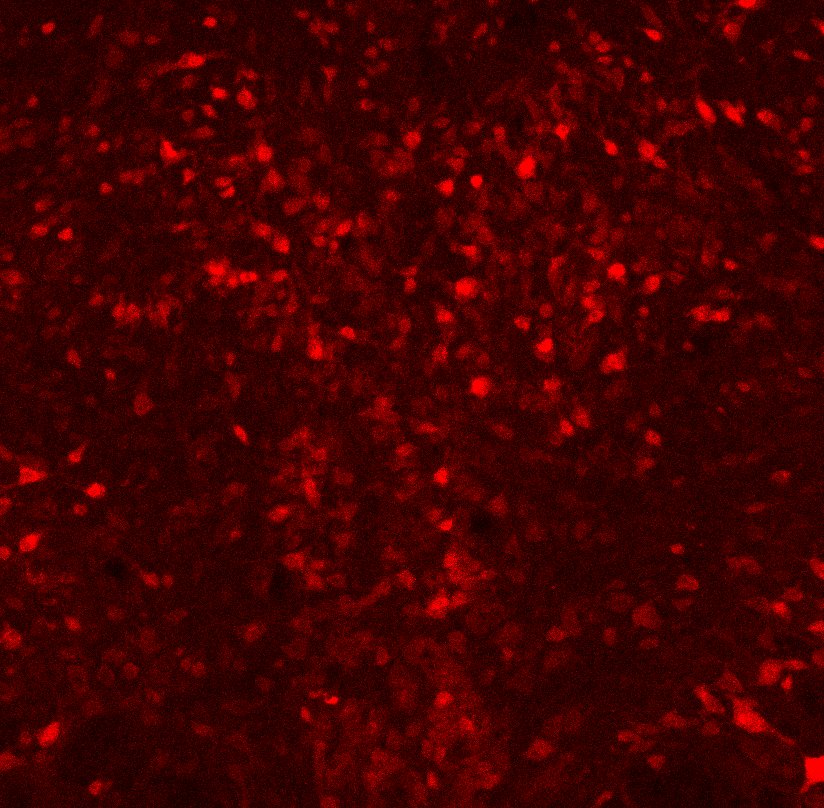

Supplement: Figure 1—figure supplement 4—source data 2. [file elife-105225-fig1-figsupp4-data2.zip › Figure 1ΓÇôFigure Supplement 4_Source Data 2/Figure 1ΓÇôFigure Supplement 4_Source Data 2_R2_NeuN_1.jpg]

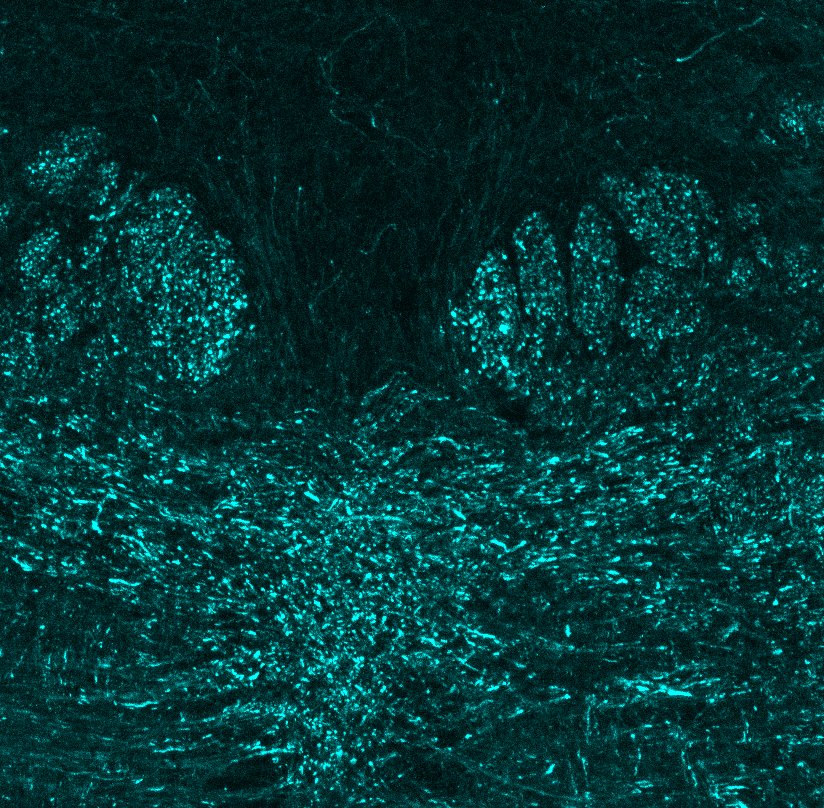

Supplement: Figure 1—figure supplement 4—source data 2. [file elife-105225-fig1-figsupp4-data2.zip › Figure 1ΓÇôFigure Supplement 4_Source Data 2/Figure 1ΓÇôFigure Supplement 4_Source Data 2_R2_NF-H_1.jpg]

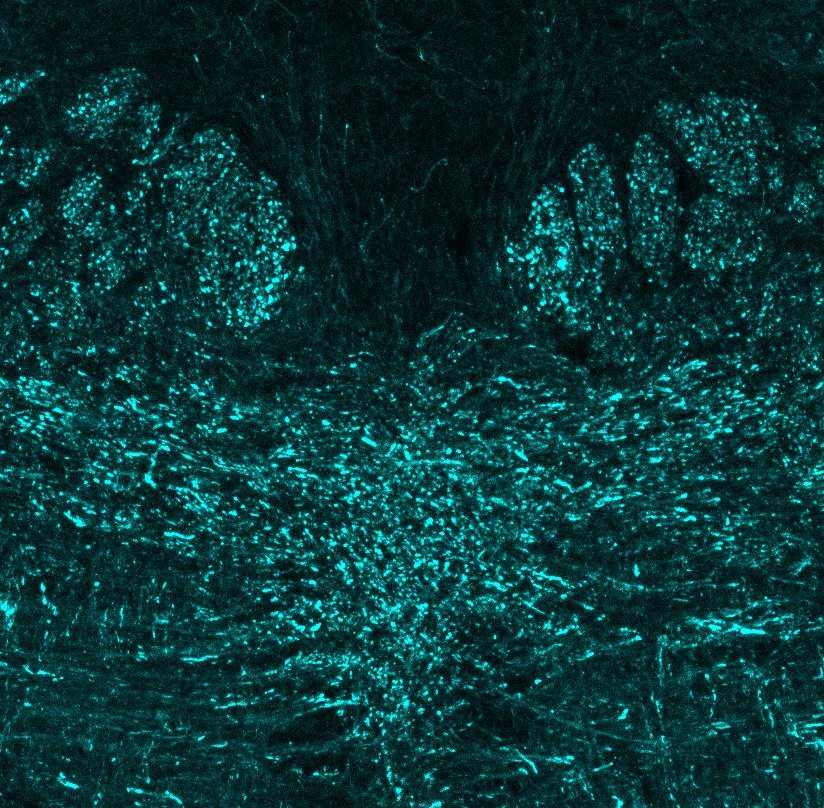

Supplement: Figure 1—figure supplement 4—source data 2. [file elife-105225-fig1-figsupp4-data2.zip › Figure 1ΓÇôFigure Supplement 4_Source Data 2/Figure 1ΓÇôFigure Supplement 4_Source Data 2_R5_NF-H_1.jpg]

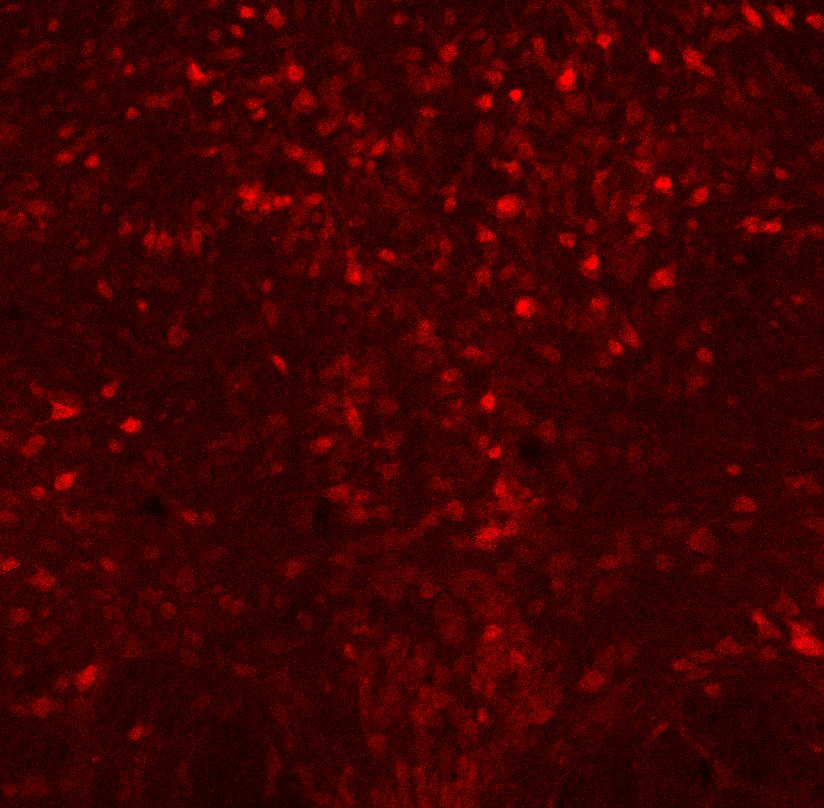

Supplement: Figure 1—figure supplement 4—source data 2. [file elife-105225-fig1-figsupp4-data2.zip › Figure 1ΓÇôFigure Supplement 4_Source Data 2/Figure 1ΓÇôFigure Supplement 4_Source Data 2_R5_NeuN_1.jpg]

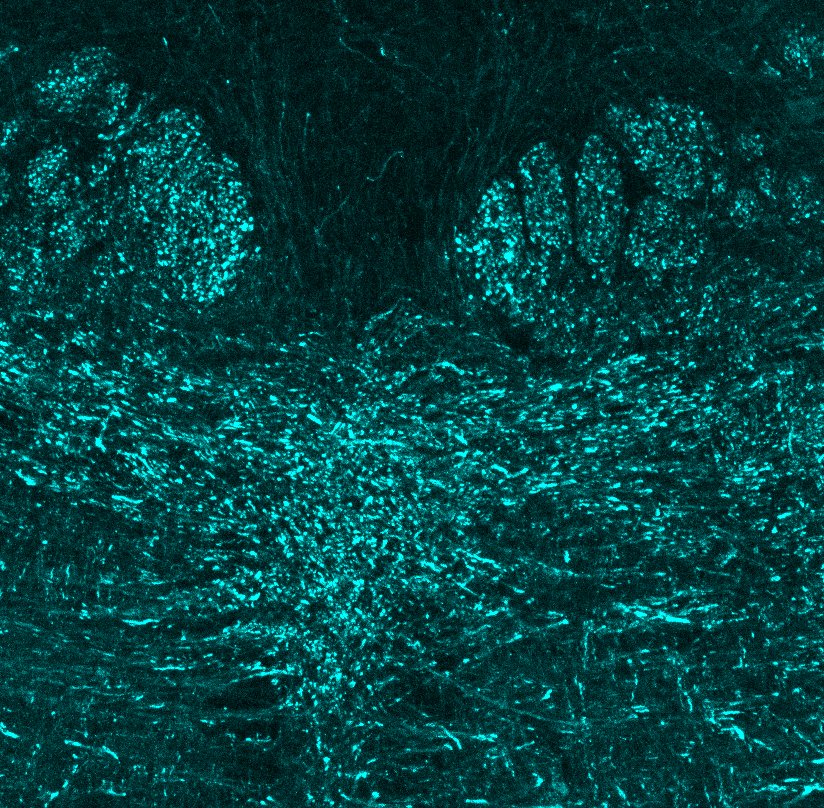

Supplement: Figure 1—figure supplement 4—source data 2. [file elife-105225-fig1-figsupp4-data2.zip › Figure 1ΓÇôFigure Supplement 4_Source Data 2/Figure 1ΓÇôFigure Supplement 4_Source Data 2_R3_NF-H_1.jpg]

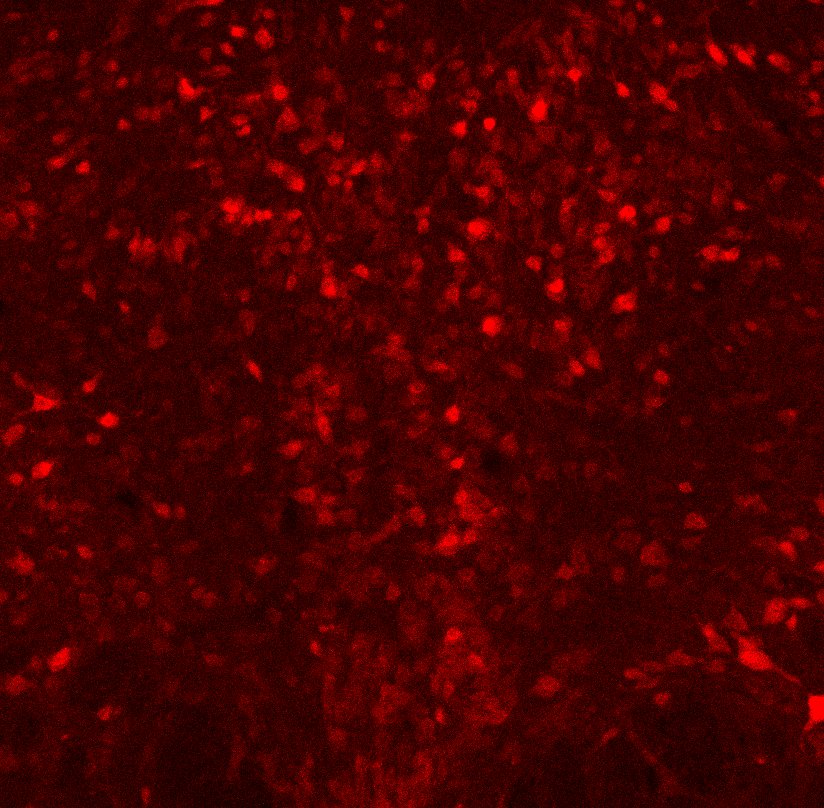

Supplement: Figure 1—figure supplement 4—source data 2. [file elife-105225-fig1-figsupp4-data2.zip › Figure 1ΓÇôFigure Supplement 4_Source Data 2/Figure 1ΓÇôFigure Supplement 4_Source Data 2_R3_NeuN_1.jpg]

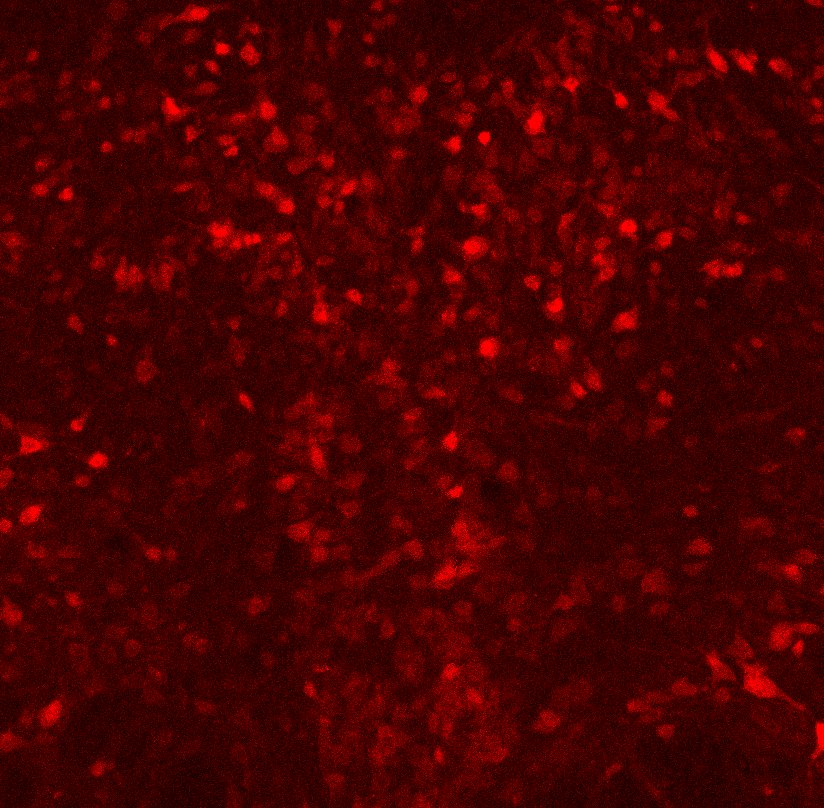

Supplement: Figure 1—figure supplement 4—source data 2. [file elife-105225-fig1-figsupp4-data2.zip › Figure 1ΓÇôFigure Supplement 4_Source Data 2/Figure 1ΓÇôFigure Supplement 4_Source Data 2_R4_NeuN_1.jpg]

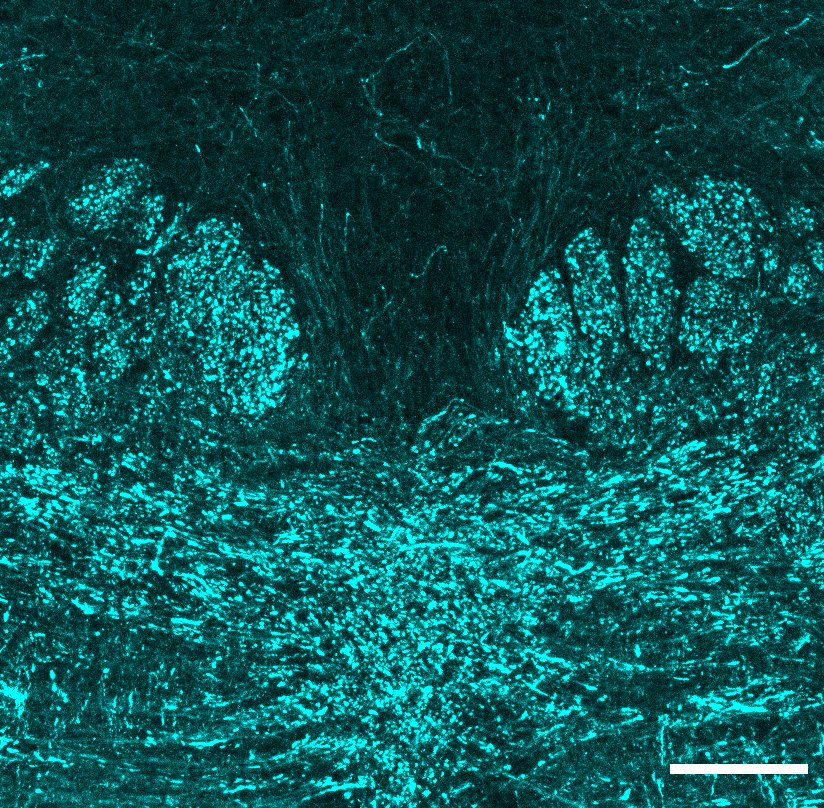

Supplement: Figure 1—figure supplement 4—source data 2. [file elife-105225-fig1-figsupp4-data2.zip › Figure 1ΓÇôFigure Supplement 4_Source Data 2/Figure 1ΓÇôFigure Supplement 4_Source Data 2_R1_NF-H_1.jpg]

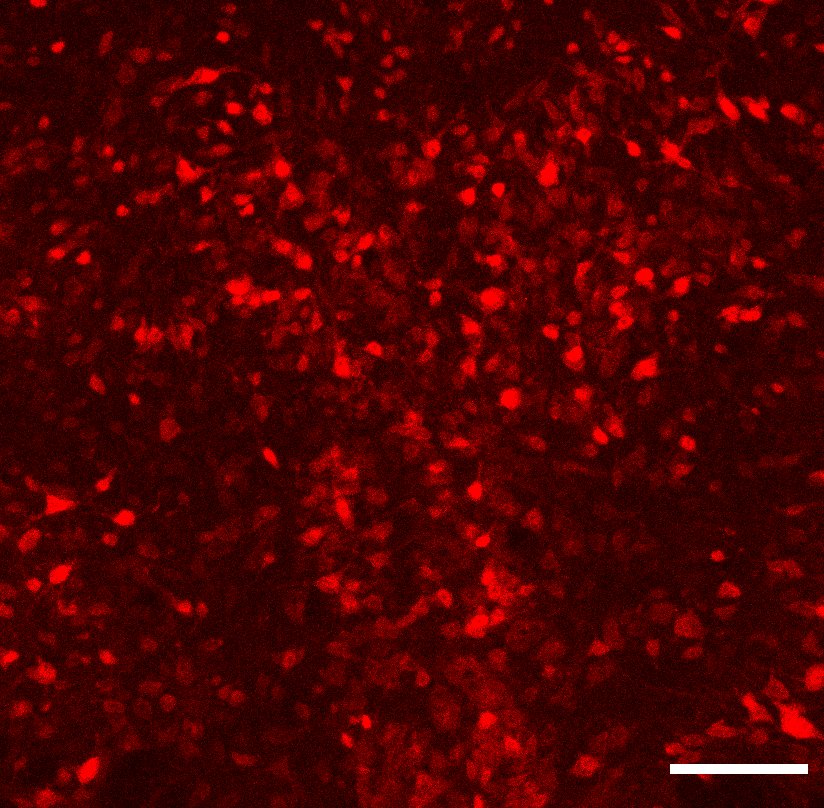

Supplement: Figure 1—figure supplement 4—source data 2. [file elife-105225-fig1-figsupp4-data2.zip › Figure 1ΓÇôFigure Supplement 4_Source Data 2/Figure 1ΓÇôFigure Supplement 4_Source Data 2_R1_NeuN_1.jpg]

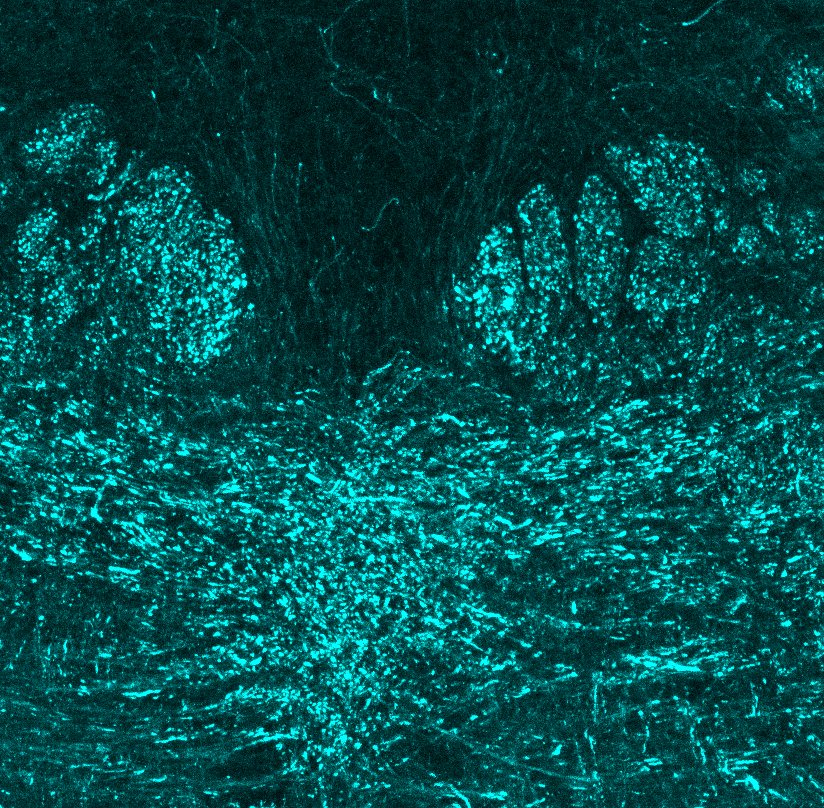

Supplement: Figure 1—figure supplement 4—source data 2. [file elife-105225-fig1-figsupp4-data2.zip › Figure 1ΓÇôFigure Supplement 4_Source Data 2/Figure 1ΓÇôFigure Supplement 4_Source Data 2_R4_NF-H_1.jpg]

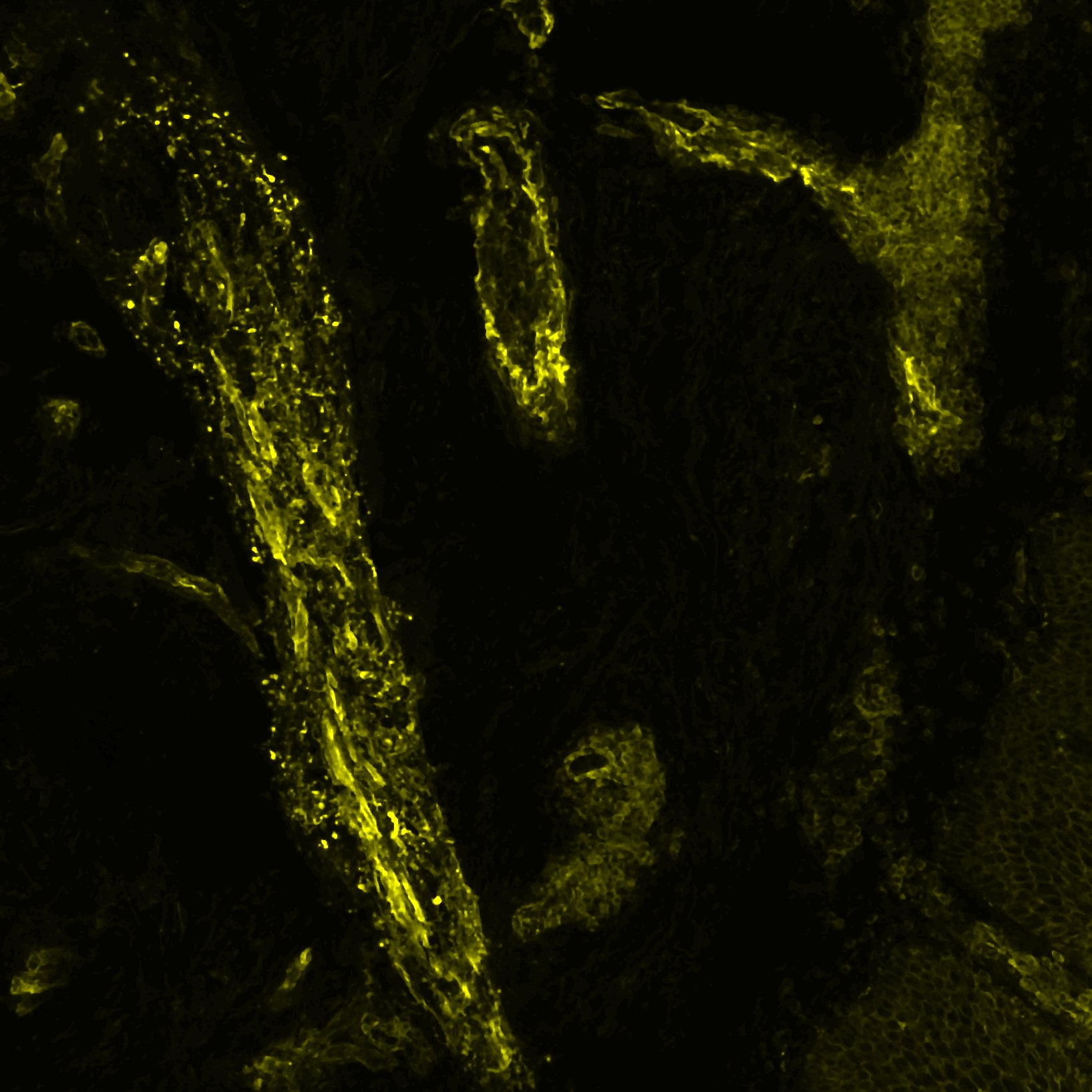

Supplement: Figure 1—figure supplement 5—source data 1. [file elife-105225-fig1-figsupp5-data1.zip › Figure 1ΓÇôFigure Supplement 5_Source Data 1/Figure 1ΓÇôFigure Supplement 5_Source Data 1_GW_#2_aSMA.jpg]

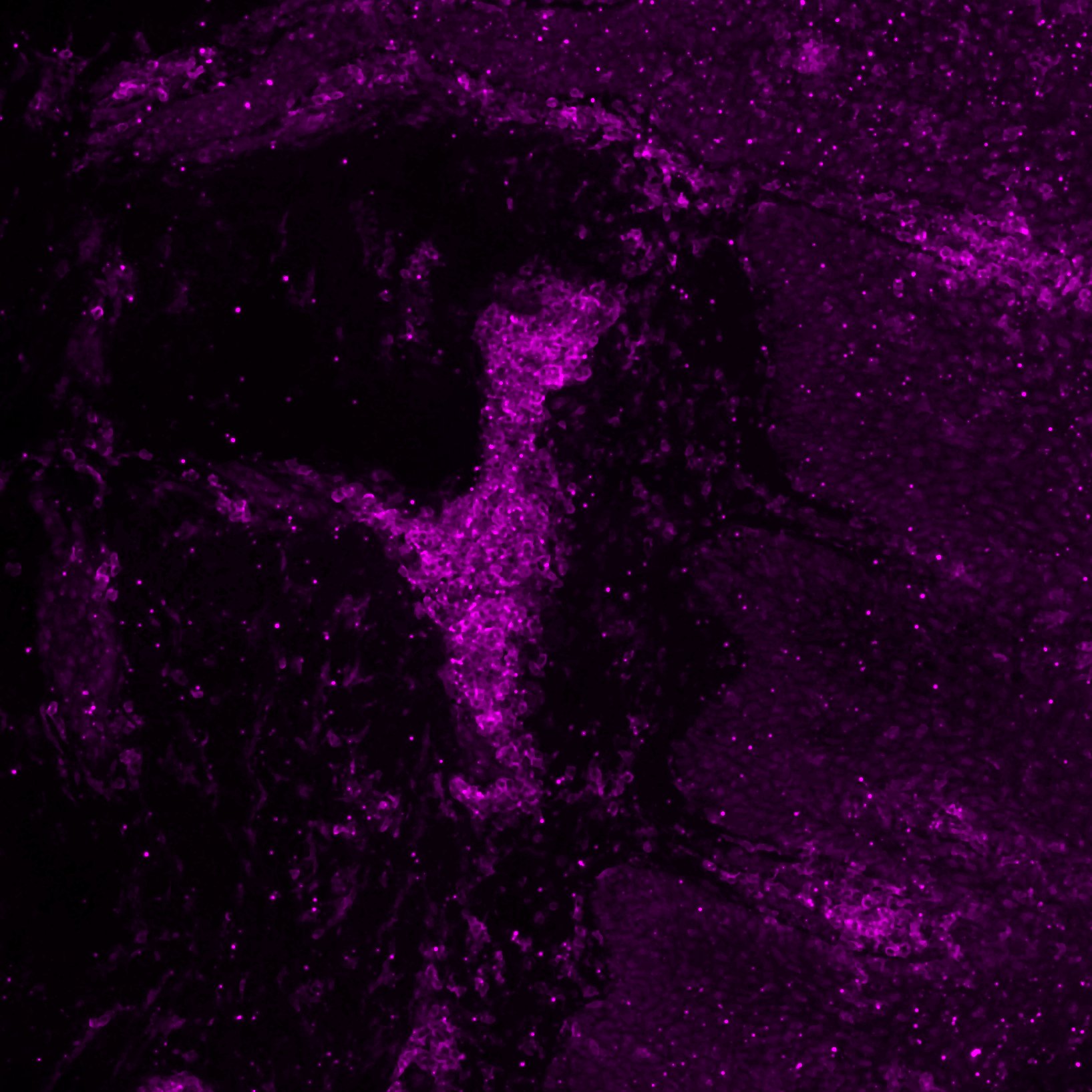

Supplement: Figure 1—figure supplement 5—source data 1. [file elife-105225-fig1-figsupp5-data1.zip › Figure 1ΓÇôFigure Supplement 5_Source Data 1/Figure 1ΓÇôFigure Supplement 5_Source Data 1_GW_#2_CD45.jpg]

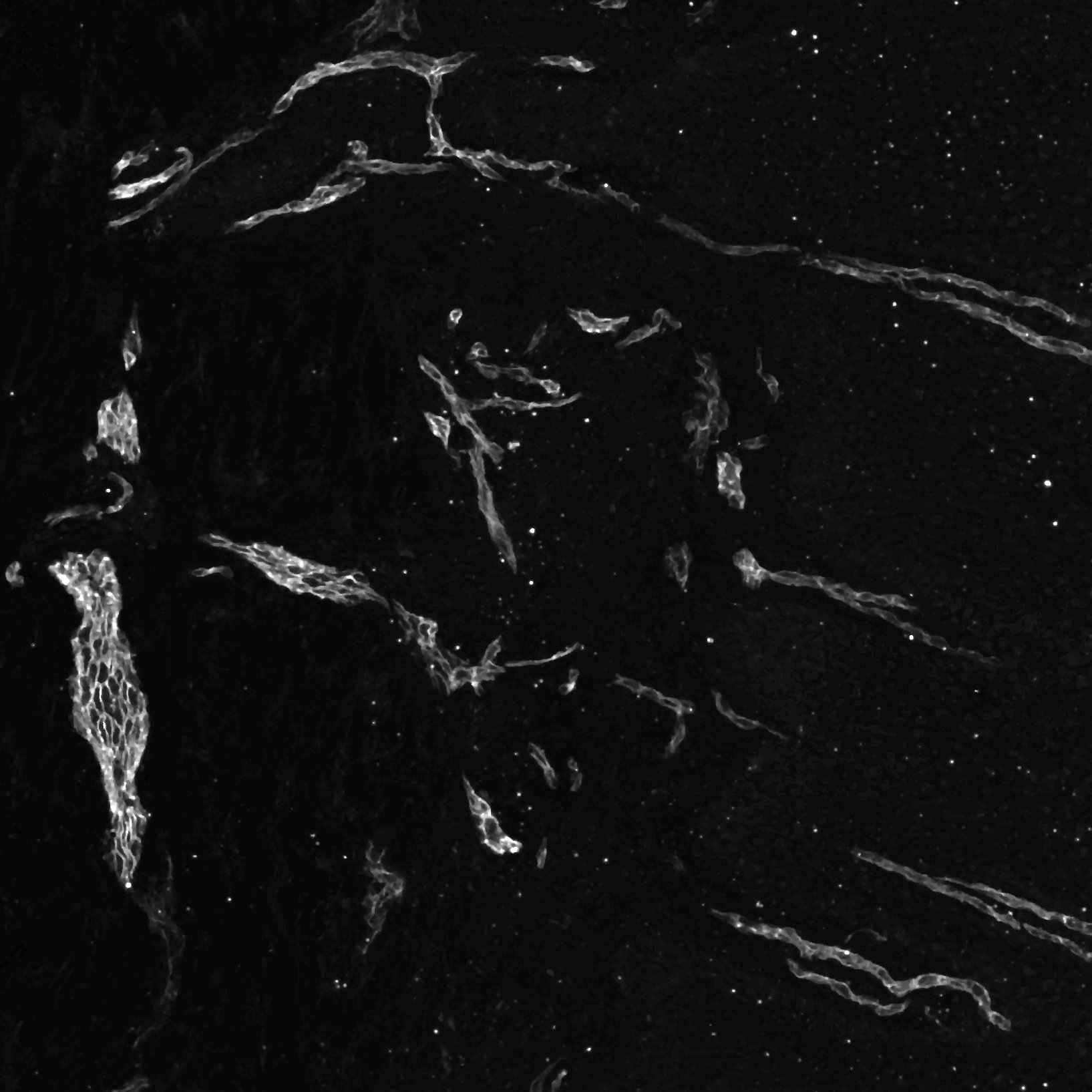

Supplement: Figure 1—figure supplement 5—source data 1. [file elife-105225-fig1-figsupp5-data1.zip › Figure 1ΓÇôFigure Supplement 5_Source Data 1/Figure 1ΓÇôFigure Supplement 5_Source Data 1_GW_#2_CD31.jpg]

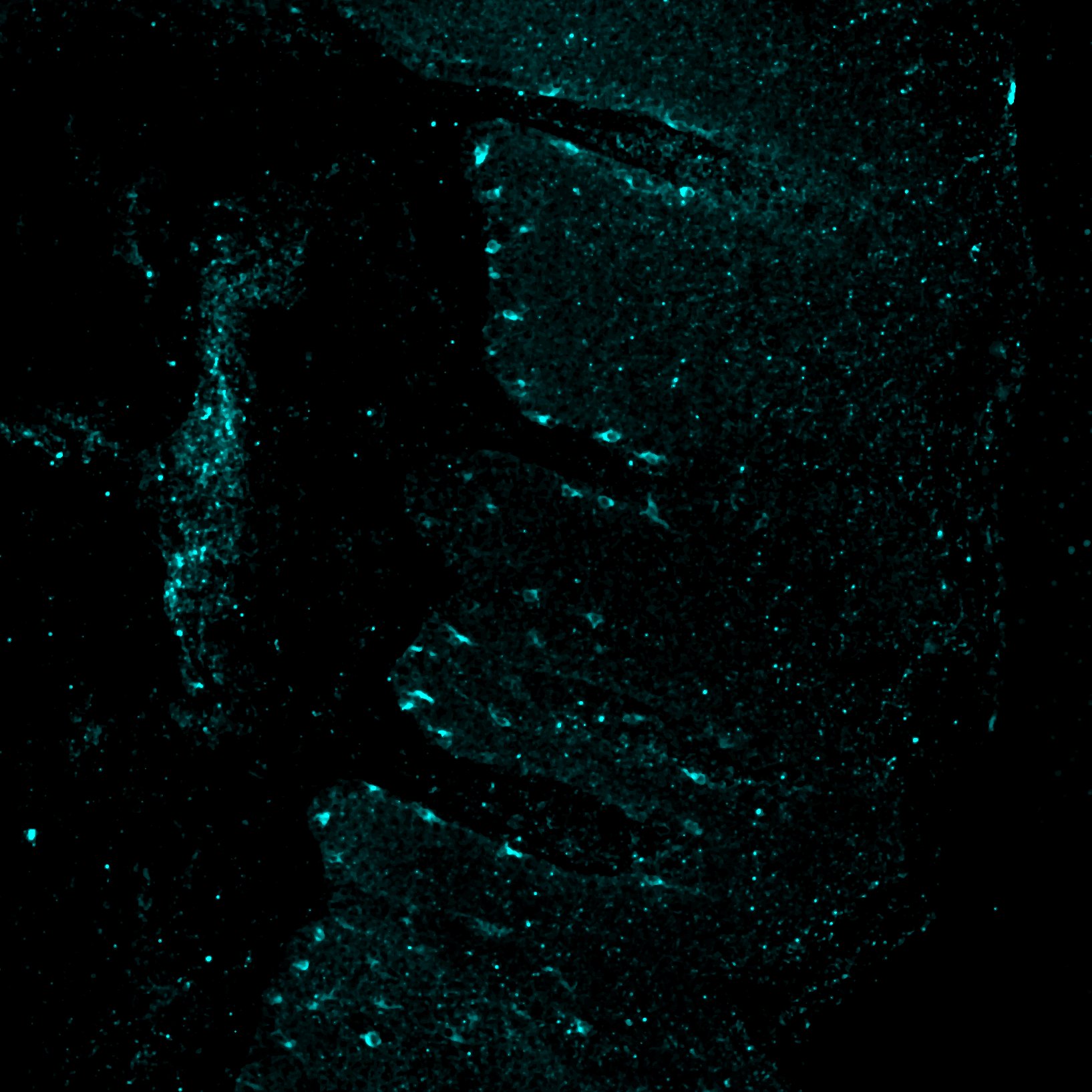

Supplement: Figure 1—figure supplement 5—source data 1. [file elife-105225-fig1-figsupp5-data1.zip › Figure 1ΓÇôFigure Supplement 5_Source Data 1/Figure 1ΓÇôFigure Supplement 5_Source Data 1_GW_#2_DCT.jpg]

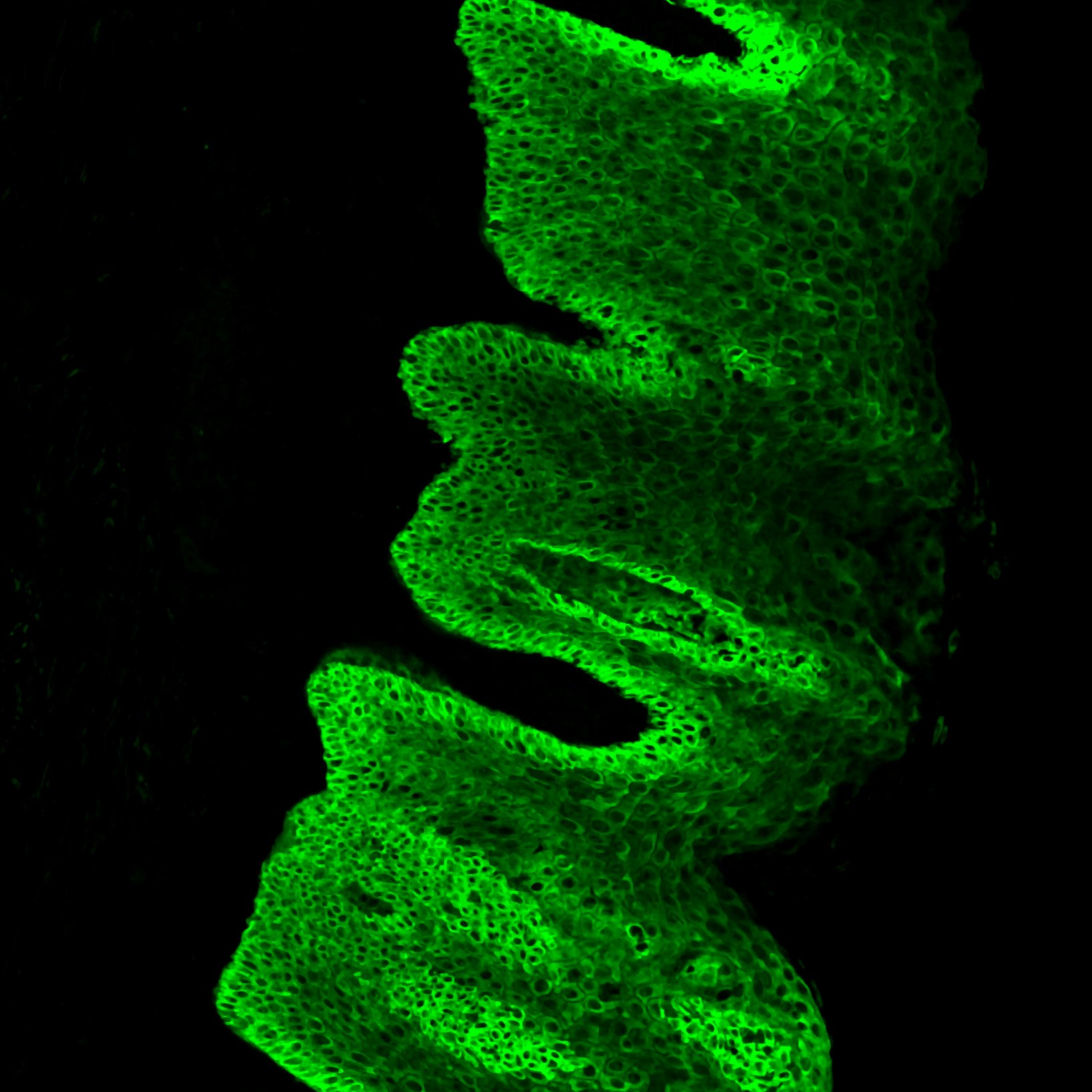

Supplement: Figure 1—figure supplement 5—source data 1. [file elife-105225-fig1-figsupp5-data1.zip › Figure 1ΓÇôFigure Supplement 5_Source Data 1/Figure 1ΓÇôFigure Supplement 5_Source Data 1_GW_#2_KRT14.jpg]

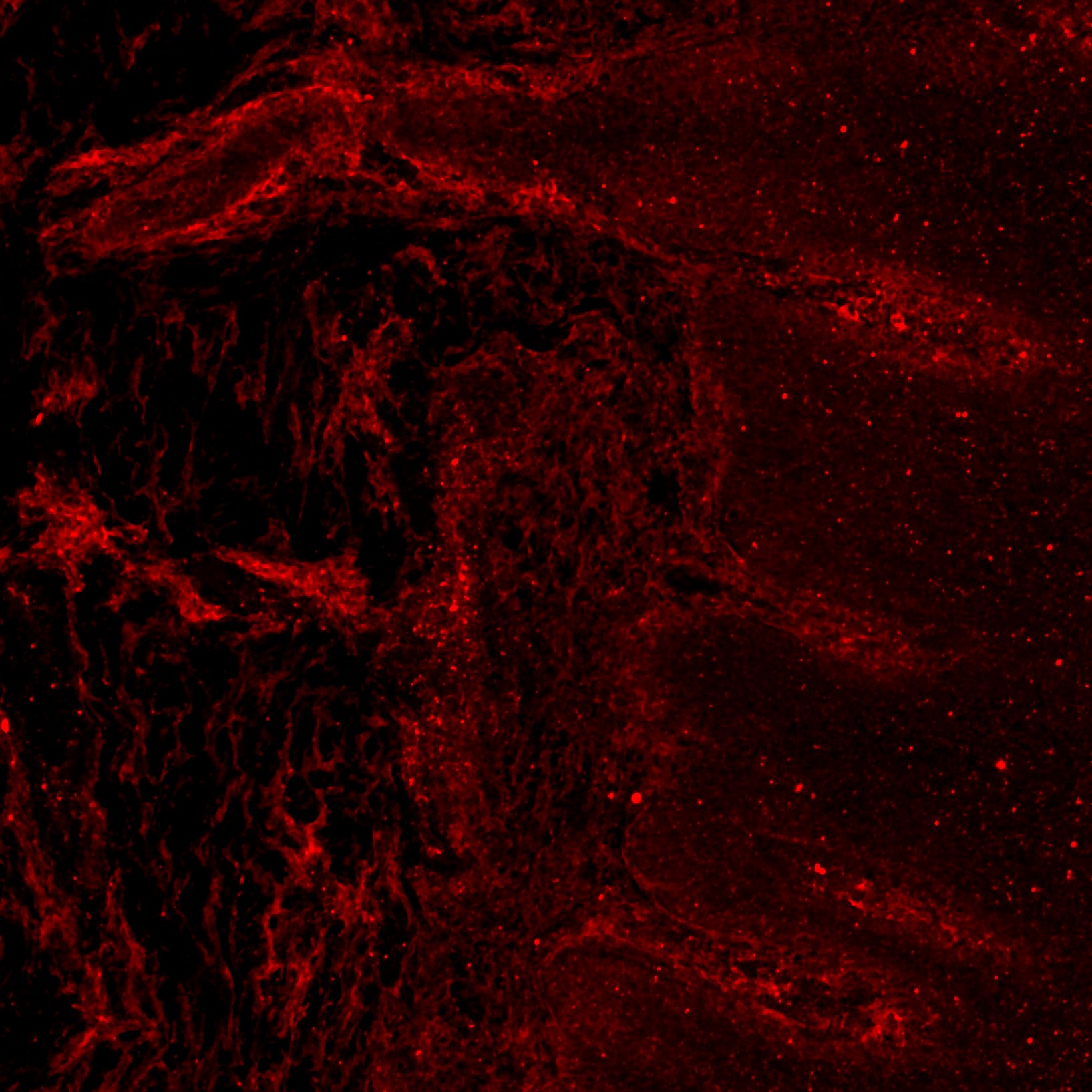

Supplement: Figure 1—figure supplement 5—source data 1. [file elife-105225-fig1-figsupp5-data1.zip › Figure 1ΓÇôFigure Supplement 5_Source Data 1/Figure 1ΓÇôFigure Supplement 5_Source Data 1_GW_#2_PDGFRa.jpg]

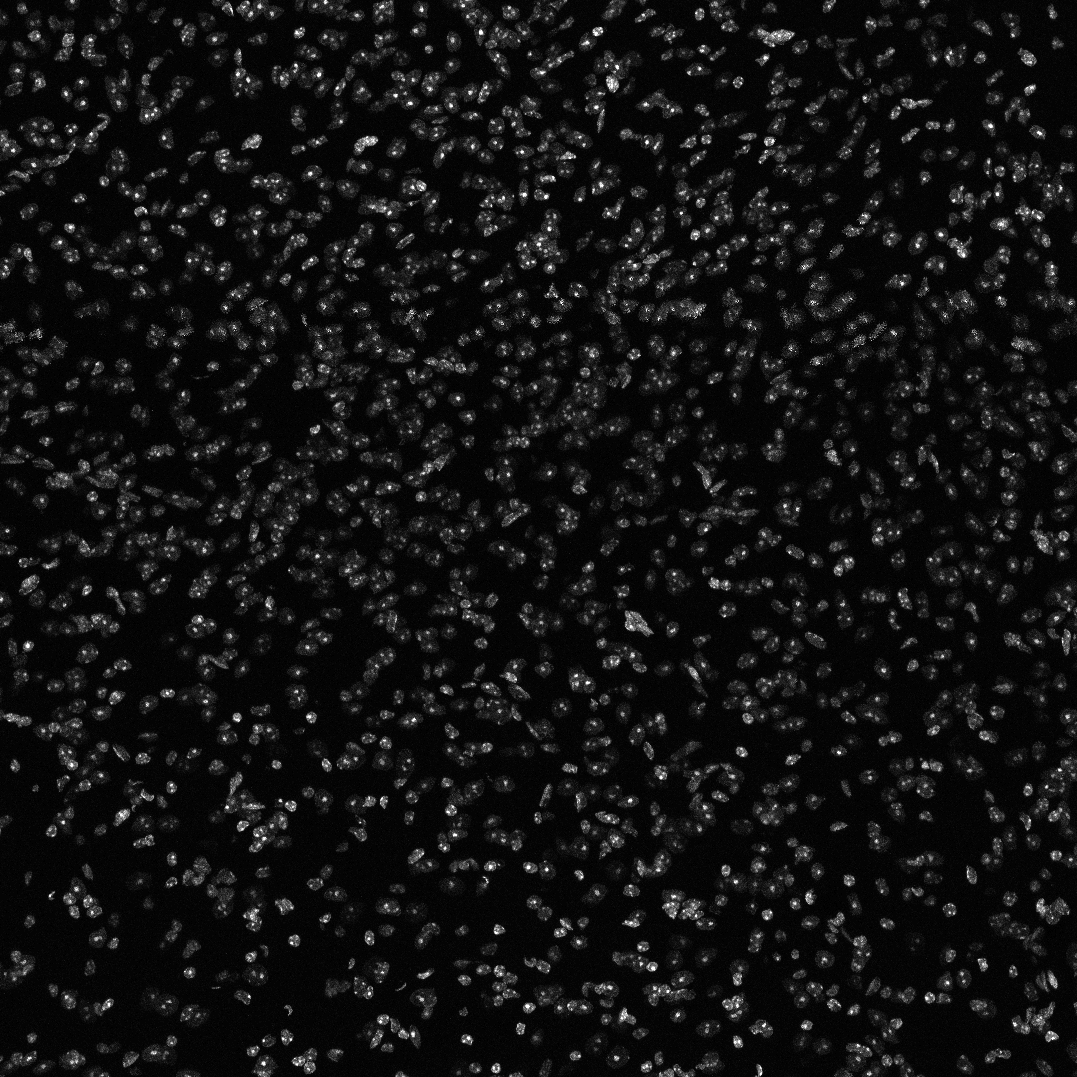

Supplement: Figure 2—source data 1. [file elife-105225-fig2-data1.zip › Figure 2_Source Data 1.tif]

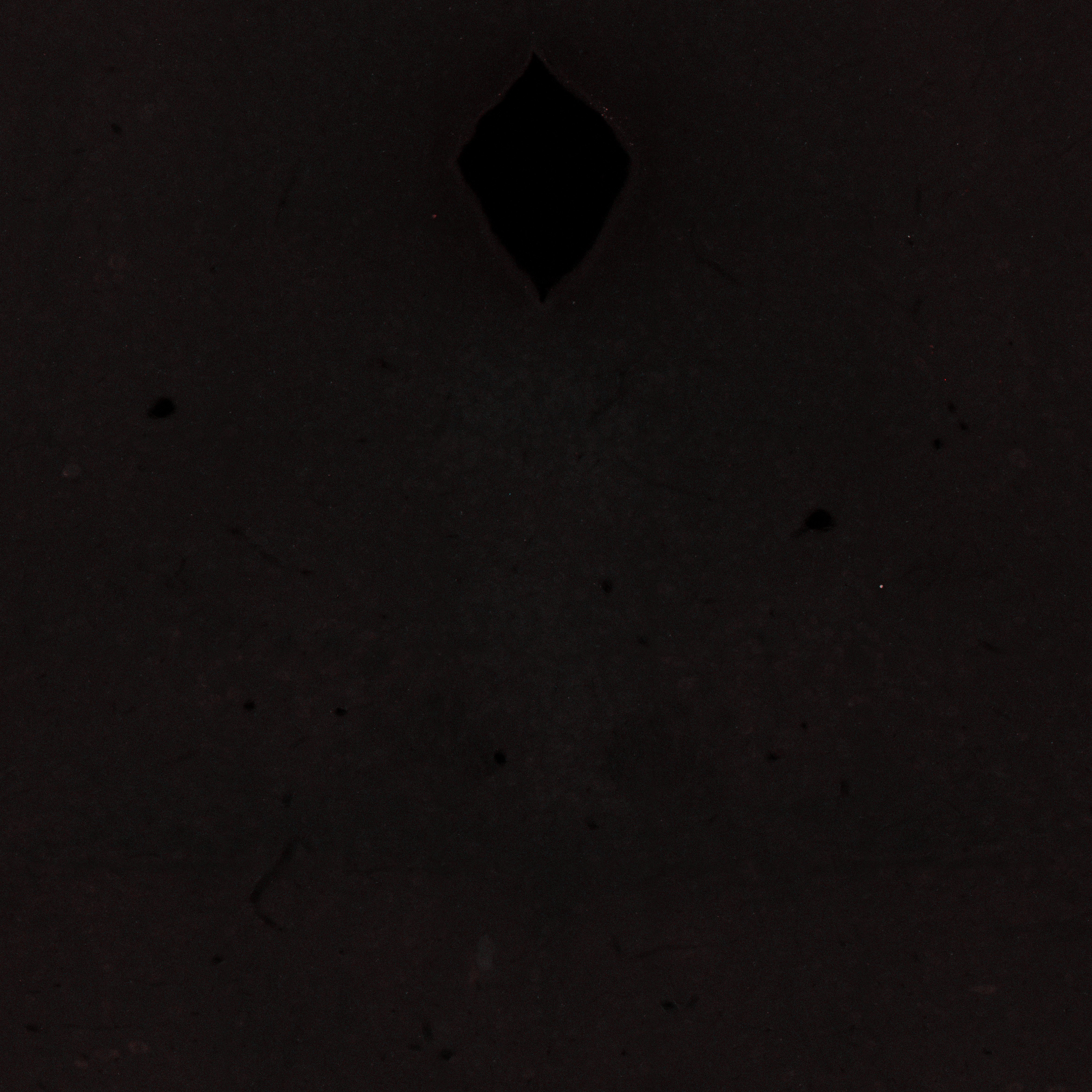

Supplement: Figure 2—figure supplement 1—source data 1. [file elife-105225-fig2-figsupp1-data1.zip › Figure 2ΓÇôFigure Supplement 1_Source Data 1/Figure 2ΓÇôFigure Supplement 1_Source Data 1_Round3 removal.jpg]

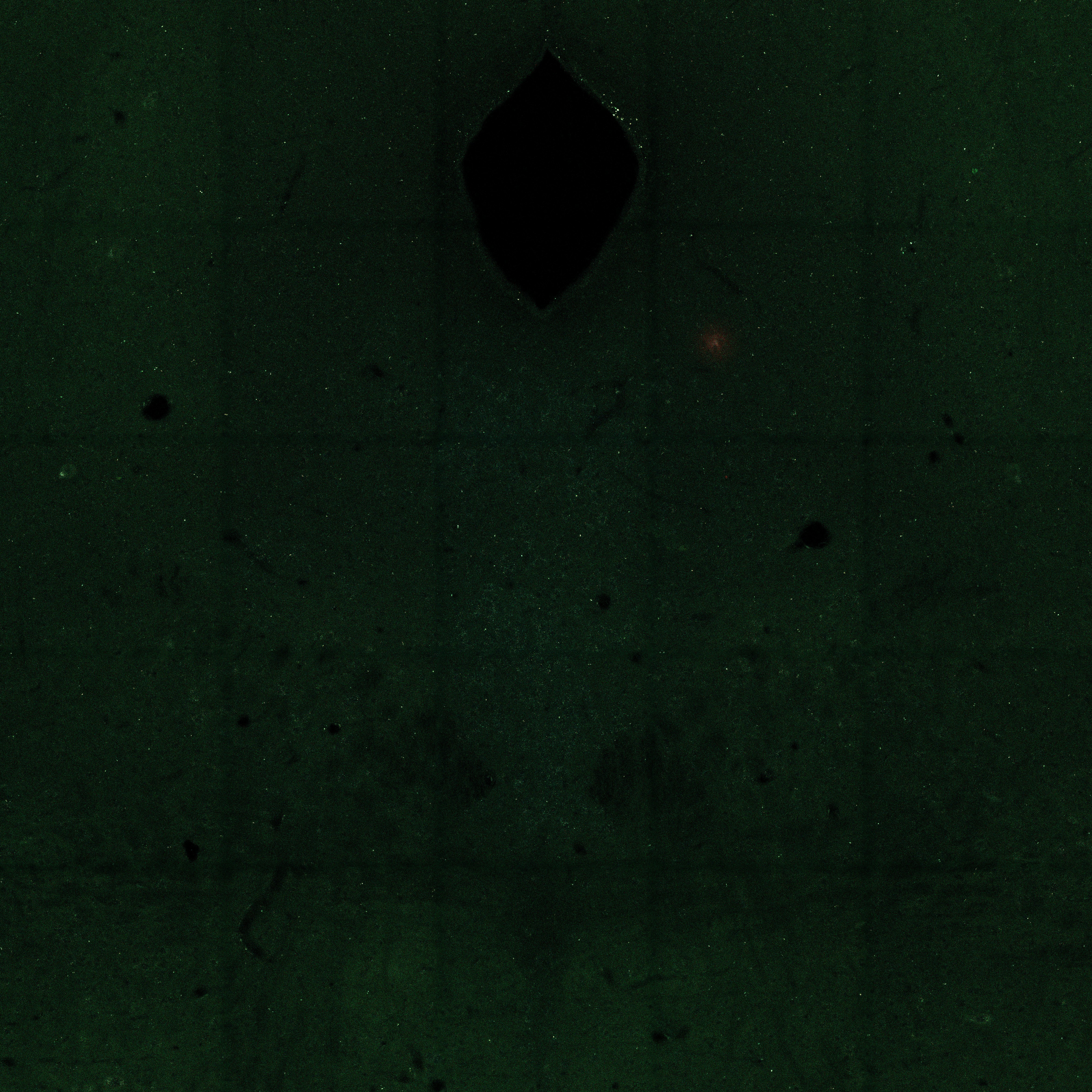

Supplement: Figure 2—figure supplement 1—source data 1. [file elife-105225-fig2-figsupp1-data1.zip › Figure 2ΓÇôFigure Supplement 1_Source Data 1/Figure 2ΓÇôFigure Supplement 1_Source Data 1_Round1 removal.jpg]

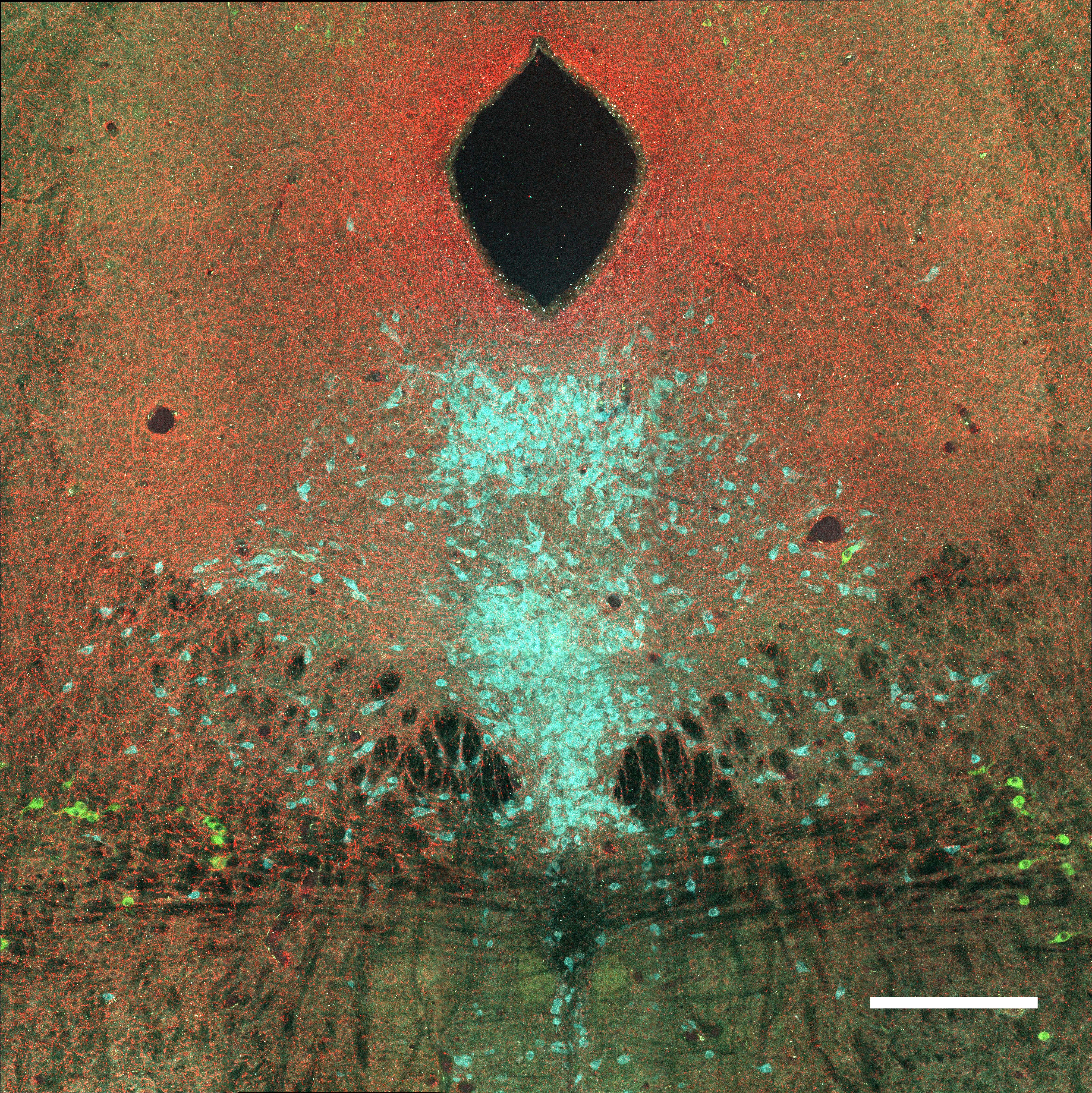

Supplement: Figure 2—figure supplement 1—source data 1. [file elife-105225-fig2-figsupp1-data1.zip › Figure 2ΓÇôFigure Supplement 1_Source Data 1/Figure 2ΓÇôFigure Supplement 1_Source Data 1_Round1.jpg]

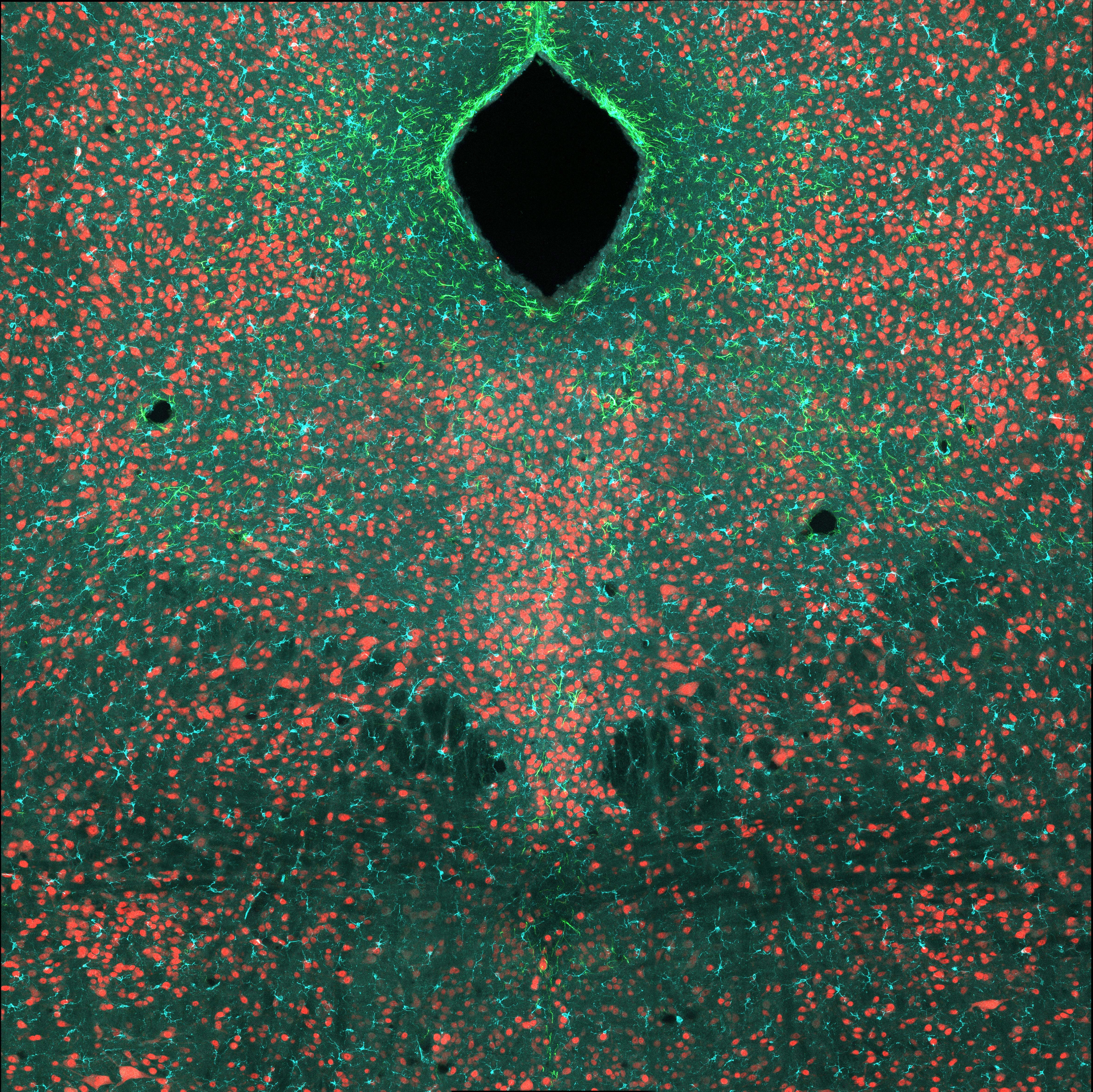

Supplement: Figure 2—figure supplement 1—source data 1. [file elife-105225-fig2-figsupp1-data1.zip › Figure 2ΓÇôFigure Supplement 1_Source Data 1/Figure 2ΓÇôFigure Supplement 1_Source Data 1_Round2.jpg]

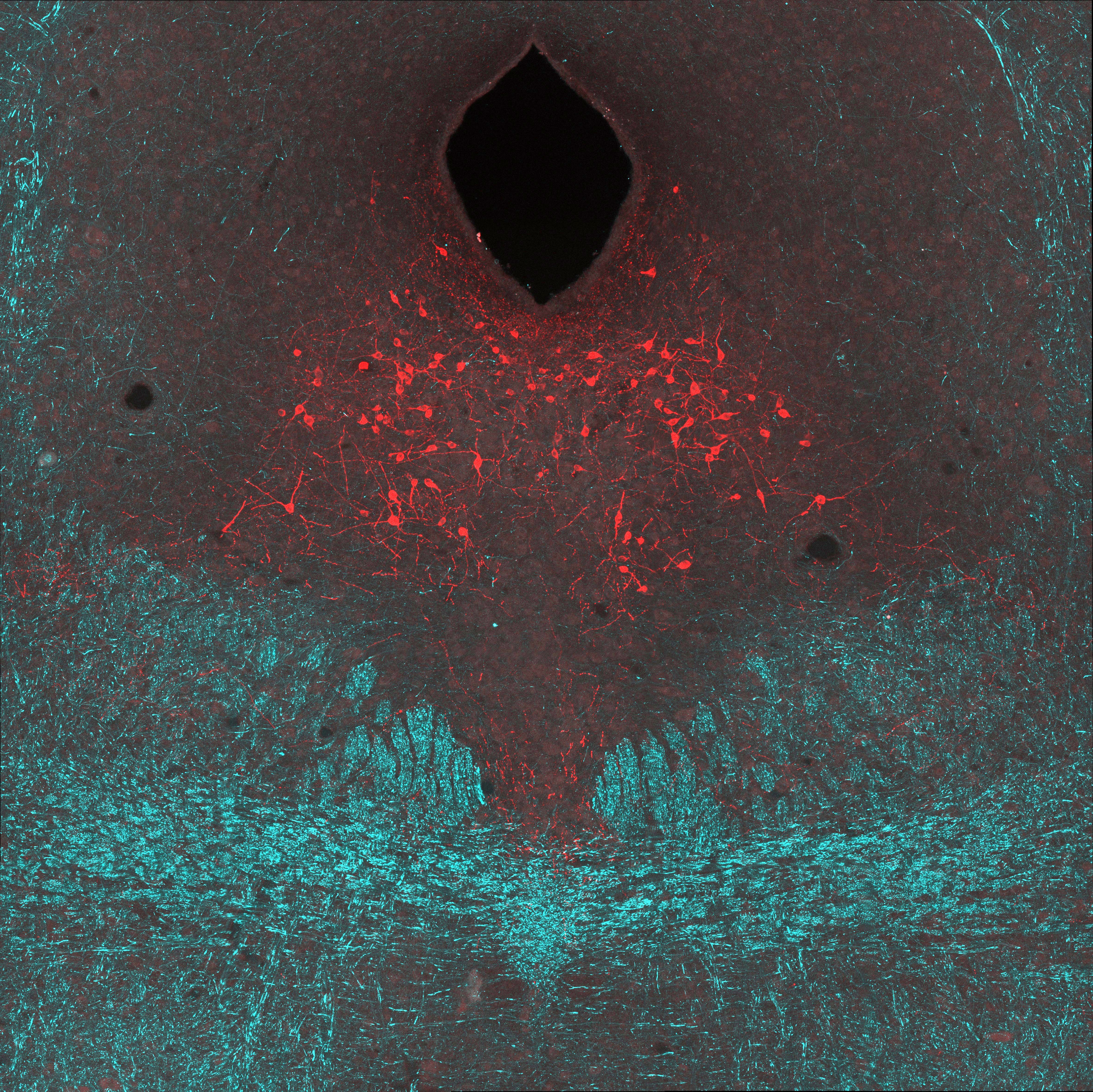

Supplement: Figure 2—figure supplement 1—source data 1. [file elife-105225-fig2-figsupp1-data1.zip › Figure 2ΓÇôFigure Supplement 1_Source Data 1/Figure 2ΓÇôFigure Supplement 1_Source Data 1_Round3.jpg]

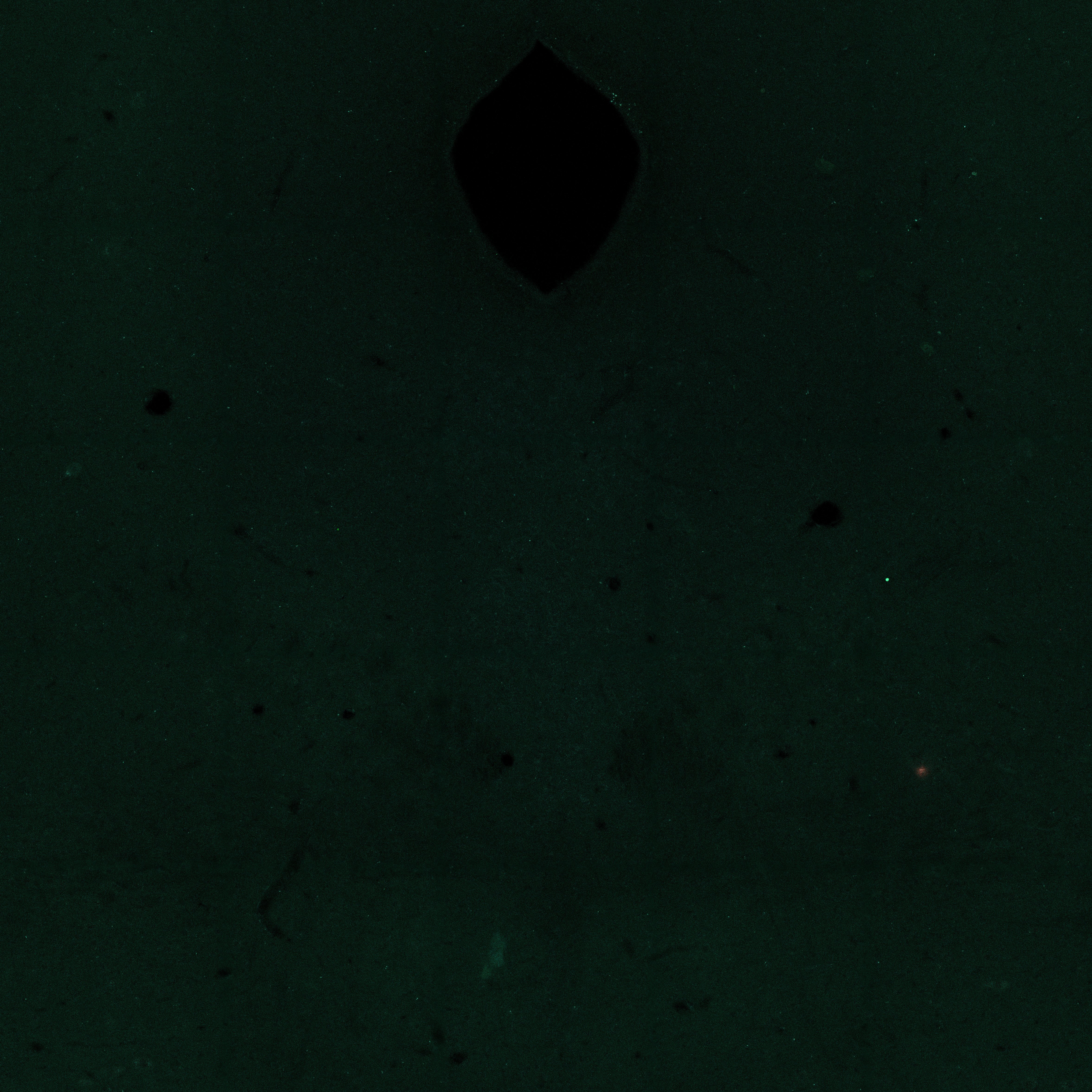

Supplement: Figure 2—figure supplement 1—source data 1. [file elife-105225-fig2-figsupp1-data1.zip › Figure 2ΓÇôFigure Supplement 1_Source Data 1/Figure 2ΓÇôFigure Supplement 1_Source Data 1_Round2 removal.jpg]

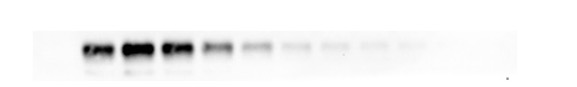

Supplement: Figure 3—source data 1. [file elife-105225-fig3-data1.zip › Figure 3_Source Data 1_2_mCherry.jpg]

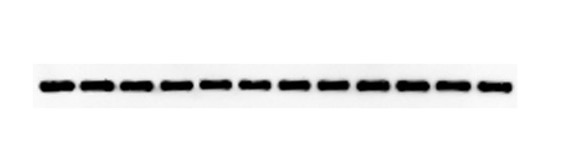

Supplement: Figure 3—source data 1. [file elife-105225-fig3-data1.zip › Figure 3_Source Data 1_3_tubulin.jpg]

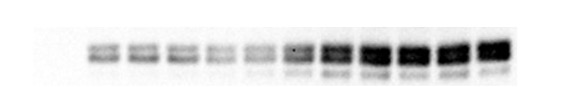

Supplement: Figure 3—source data 1. [file elife-105225-fig3-data1.zip › Figure 3_Source Data 1_GFP.jpg]

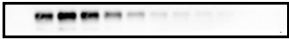

Supplement: Figure 3—source data 2. [file elife-105225-fig3-data2.zip › Figure 3_Source Data 2_2_mCherry.pdf]

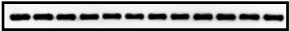

Supplement: Figure 3—source data 2. [file elife-105225-fig3-data2.zip › Figure 3_Source Data 2_3_tubulin.pdf]

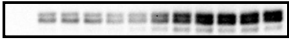

Supplement: Figure 3—source data 2. [file elife-105225-fig3-data2.zip › Figure 3_Source Data 2_GFP.pdf]

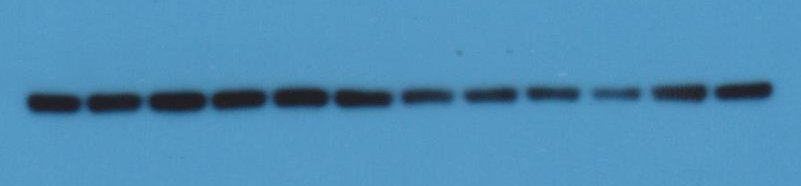

Supplement: Figure 3—figure supplement 1—source data 1. [file elife-105225-fig3-figsupp1-data1.zip › Figure 3ΓÇôFigure Supplement 1_Source Data 1_2_tubulin.jpg]

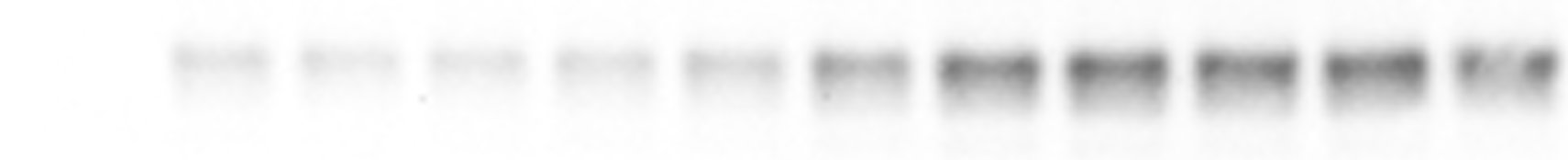

Supplement: Figure 3—figure supplement 1—source data 1. [file elife-105225-fig3-figsupp1-data1.zip › Figure 3ΓÇôFigure Supplement 1_Source Data 1_3_mCherry.jpg]

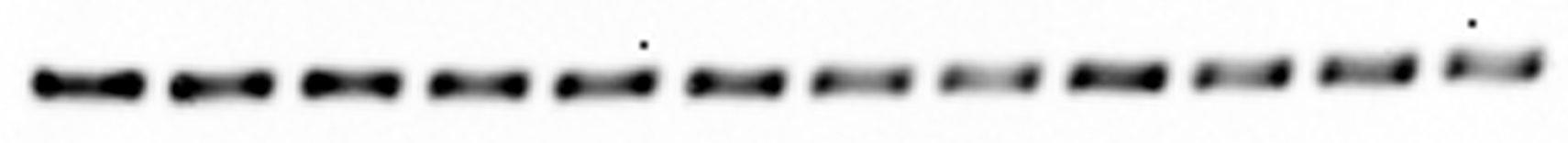

Supplement: Figure 3—figure supplement 1—source data 1. [file elife-105225-fig3-figsupp1-data1.zip › Figure 3ΓÇôFigure Supplement 1_Source Data 1_4_tubulin.jpg]

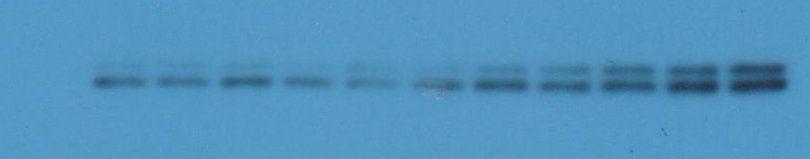

Supplement: Figure 3—figure supplement 1—source data 1. [file elife-105225-fig3-figsupp1-data1.zip › Figure 3ΓÇôFigure Supplement 1_Source Data 1_GFP.jpg]

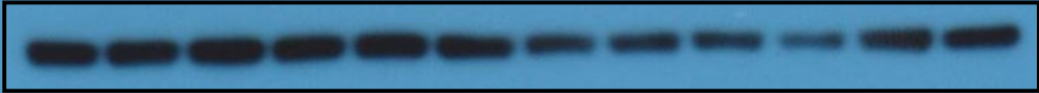

Supplement: Figure 3—figure supplement 1—source data 2. [file elife-105225-fig3-figsupp1-data2.zip › Figure 3ΓÇôFigure Supplement 1_Source Data 2_2_tubulin.pdf]

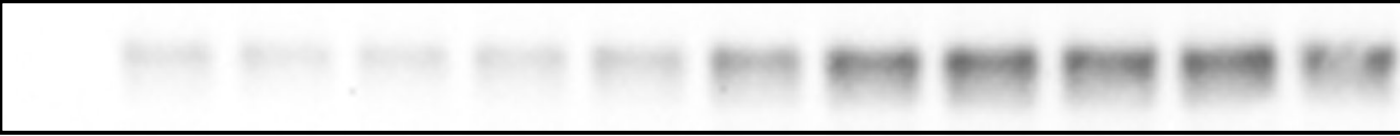

Supplement: Figure 3—figure supplement 1—source data 2. [file elife-105225-fig3-figsupp1-data2.zip › Figure 3ΓÇôFigure Supplement 1_Source Data 2_3_mCherry.pdf]

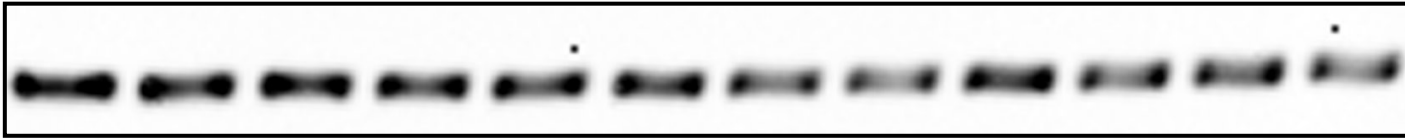

Supplement: Figure 3—figure supplement 1—source data 2. [file elife-105225-fig3-figsupp1-data2.zip › Figure 3ΓÇôFigure Supplement 1_Source Data 2_4_tubulin.pdf]

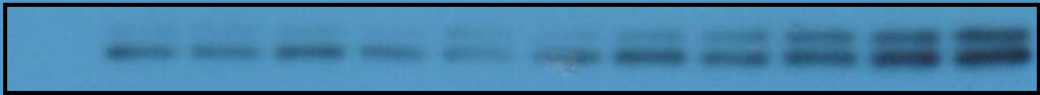

Supplement: Figure 3—figure supplement 1—source data 2. [file elife-105225-fig3-figsupp1-data2.zip › Figure 3ΓÇôFigure Supplement 1_Source Data 2_GFP.pdf]
