# Supplementary material for: Modular DNA barcoding of nanobodies enables multiplexed in situ protein imaging and high-throughput biomolecule detection
Source: eLife. 2025 Jul 22;14:RP105225. doi: 10.7554/eLife.105225 (PMC12283080; doi:10.7554/eLife.105225)
Supplement: Supplementary file 2. [file elife-105225-supp2.docx]

**Supplementary File 2. Antibodies and Nb-DNA oligos (or Nb-SS-DNA oligos).**

| **Epitope** | **Host** | **Nb-DNA oligos (or Nb-SS-DNA oligos)** |
| --- | --- | --- |
| PDGFRα | Rabbit | TP897-B1 I1 |
| KRT14 | Rabbit | TP897-B3 I1 |
| DCT | Rabbit | TP897-B4 I1 |
| CD31 | Mouse (IgG1) | TP1107-B9 I1 |
| αSMA | Rabbit | TP897-B10 I1 |
| CD45 | Mouse (IgG1) | TP1107-B13 I1 |
| MAP2 | Rabbit | TP897-B1 I1 |
| NeuN | Rabbit | TP897-B1 I1, TP897-SS-B15 I1 |
| TPH2 | Rabbit | TP897-B5 I1, TP897-SS-B5 I1 |
| TH | Rabbit | TP897-B1 I1, TP897-SS-B14 I1 |
| nNOS | Rabbit | TP897-B5 I1, TP897-SS-B10 I1 |
| GFAP | Rabbit | TP897-B5 I1, TP897-SS-B13 I1 |
| Iba1 | Rabbit | TP897-B4 I1, TP897-SS-B13 I1 |
| DDC | Rabbit | TP897-B4 I1, TP897-SS-B14 I1 |
| NF-H | Rabbit | TP897-B4 I1, TP897-SS-B17 I1 |
| TMEM119 | Rabbit | TP897-B1 I1, TP897-SS-B15 I1 |
| GABA | Rabbit | TP897-B1 I1, TP897-SS-B2 I1 |
| Orexin A | Rabbit | TP897-B1 I1, TP897-SS-B9 I1 |
| 5-HT | Rabbit | TP897-B1 I1, TP897-SS-B4 I1 |
| α-tubulin | Mouse (IgG1) | TP1107-SS-qbc. 1, TP1107-SS-sbc. 1 |
| GFP | Rabbit | TP897-SS-qbc. 2, TP897-SS -sbc. 2 |
| mCherry | Rabbit | TP897-SS-qbc. 3, TP897-SS-sbc. 3 |
| Human IgG | Rabbit | TP897-SS-qbc. 2, TP897-SS-sbc. 4 |
| HBsAg (capture) | Goat |  |
| HBsAg (detection) | Mouse (IgG1) | TP1107-SS-qbc. 3, TP1107-SS-sbc. 3 |
| HBeAg (capture) | Mouse (IgG2a) |  |
| HBeAg (detection) | Mouse (IgG1) | TP1107-SS-qbc. 2, TP1107-SS-sbc. 2 |
| phospho-p38α (T180/Y182) (capture) | Mouse |  |
| phospho-p38α (T180/Y182) (detection) | Rabbit | TP897-SS-qbc. 2, TP897-SS-sbc. 2 |
| phospho-ERK1 (T202/Y204)/ERK2 (T185/Y187) (capture) | Mouse |  |
| phospho-ERK1 (T202/Y204)/ERK2 (T185/Y187) (detection) | Rabbit | TP897-SS-qbc. 3, TP897-SS-sbc. 3 |
| phospho-JNK Pan Specific (capture) | Mouse |  |
| phospho-JNK Pan Specific (detection) | Rabbit | TP897-SS-qbc. 4, TP897-SS-sbc. 4 |
| phospho-AMPKα1 (T183) (capture) | Goat |  |
| phospho-AMPKα1 (T183) (detection) | Rabbit | TP897-SS-qbc. 5, TP897-SS-sbc. 5 |
| phospho-CREB (S133) (capture) | Goat |  |
| phospho-CREB (S133) (detection) | Rabbit | TP897-SS-qbc. 6, TP897-SS-sbc. 6 |
| phospho-Src (Y419) (capture) | Goat |  |
| phospho-Src (Y419) (detection) | Rabbit | TP897-SS-qbc. 7, TP897-SS-sbc. 7 |
| phospho-Akt (S473) (capture) | Rabbit |  |
| phospho-Akt (S473) (detection) | Rabbit | TP1107-SS-qbc. 1, TP1107-SS-sbc. 1 |
