# Supplementary material for: Modular DNA barcoding of nanobodies enables multiplexed in situ protein imaging and high-throughput biomolecule detection
Source: eLife. 2025 Jul 22;14:RP105225. doi: 10.7554/eLife.105225 (PMC12283080; doi:10.7554/eLife.105225)
Supplement: Supplementary file 3. [file elife-105225-supp3.docx]

**Supplementary File 3. Detailed sequences and modifications of DNA oligos.**

| **Name** | **Sequence (5’ to 3’)** | **Modifications** |
| --- | --- | --- |
| **B1 I1** | ATATAGCATTCTTTCTTGAGGAGGGCAGCAAACGGGAAGAG | 5’ DBCO or 5’ Amino C6 |
| **B2 I1** | ATATAAGCTCAGTCCATCCTCGTAAATCCTCATCAATCATC | 5’ DBCO or 5’ Amino C6 |
| **B3 I1** | ATATAAAAGTCTAATCCGTCCCTGCCTCTATATCTCCACTC | 5’ DBCO or 5’ Amino C6 |
| **B4 I1** | ATATACACATTTACAGACCTCAACCTACCTCCAACTCTCAC | 5’ DBCO or 5’ Amino C6 |
| **B5 I1** | ATATACACTTCATATCACTCACTCCCAATCTCTATCTACCC | 5’ DBCO or 5’ Amino C6 |
| **B9 I1** | ATATACACGTATCTACTCCACTCTCAGCACACTCCCAACCC | 5’ DBCO or 5’ Amino C6 |
| **B10 I1** | ATATACCTCAAGATACTCCTCTACCTACTCGACTACCCTAG | 5’ DBCO or 5’ Amino C6 |
| **B13 I1** | ATATAAGGTAACGCCTTCCTGCTTTATGCTCAACATACAAC | 5’ DBCO or 5’ Amino C6 |
| **B14 I1** | ATATAAATGTCAATAGCGAGCGACCCTATATTTCTGCACAG | 5’ DBCO or 5’ Amino C6 |
| **B15 I1** | ATATACAGATTAACACACCACAAGGTATCTCGAACACTCTC | 5’ DBCO or 5’ Amino C6 |
| **B17 I1** | ATATACGATTGTTTGTTGTGGACGCATGCTAATCGGATGAG | 5’ DBCO or 5’ Amino C6 |
| **B1 Amplifier H1** | CGTAAAGGAAGACTCTTCCCGTTTGCTGCCCTCCTCGCATTCTTTCTTGAGGAGGGCAGCAAACGGGAAGAG | 5' Alexa Fluor 546 or 5’ Cy3 |
| **B1 Amplifier H2** | GAGGAGGGCAGCAAACGGGAAGAGTCTTCCTTTACGCTCTTCCCGTTTGCTGCCCTCCTCAAGAAAGAATGC | 3' Alexa Fluor 546 or 3’ Cy3 |
| **B2 Amplifier H1** | GGCGGTTTACTGGATGATTGATGAGGATTTACGAGGAGCTCAGTCCATCCTCGTAAATCCTCATCAATCATC | 5' Alexa Fluor 594 |
| **B2 Amplifier H2** | CCTCGTAAATCCTCATCAATCATCCAGTAAACCGCCGATGATTGATGAGGATTTACGAGGATGGACTGAGCT | 3' Alexa Fluor 594 |
| **B3 Amplifier H1** | CGGGTTAAAGTTGAGTGGAGATATAGAGGCAGGGACAAAGTCTAATCCGTCCCTGCCTCTATATCTCCACTC | 5' Alexa Fluor 488 |
| **B3 Amplifier H2** | GTCCCTGCCTCTATATCTCCACTCAACTTTAACCCGGAGTGGAGATATAGAGGCAGGGACGGATTAGACTTT | 3' Alexa Fluor 488 |
| **B4 Amplifier H1** | GAAGCGAATATGGTGAGAGTTGGAGGTAGGTTGAGGCACATTTACAGACCTCAACCTACCTCCAACTCTCAC | 5' Alexa Fluor 647 or 5’ Cy5 |
| **B4 Amplifier H2** | CCTCAACCTACCTCCAACTCTCACCATATTCGCTTCGTGAGAGTTGGAGGTAGGTTGAGGTCTGTAAATGTG | 3' Alexa Fluor 647 or 3’ Cy5 |
| **B5 Amplifier H1** | ATTGGATTTGTAGGGTAGATAGAGATTGGGAGTGAGCACTTCATATCACTCACTCCCAATCTCTATCTACCC | 5' Alexa Fluor 488 |
| **B5 Amplifier H2** | CTCACTCCCAATCTCTATCTACCCTACAAATCCAATGGGTAGATAGAGATTGGGAGTGAGTGATATGAAGTG | 3' Alexa Fluor 488 |
| **B9 Amplifier H1** | CCACTCTCAGCACACTCCCAACCCTACTACAAGCTCGGGTTGGGAGTGTGCTGAGAGTGGAGTAGATACGTG | 5' Alexa Fluor 647 |
| **B9 Amplifier H2** | GAGCTTGTAGTAGGGTTGGGAGTGTGCTGAGAGTGGCACGTATCTACTCCACTCTCAGCACACTCCCAACCC | 3' Alexa Fluor 647 |
| **B10 Amplifier H1** | CCTCTACCTACTCGACTACCCTAGCCGTAACTTCACCTAGGGTAGTCGAGTAGGTAGAGGAGTATCTTGAGG | 5' Alexa Fluor 488 |
| **B10 Amplifier H2** | GTGAAGTTACGGCTAGGGTAGTCGAGTAGGTAGAGGCCTCAAGATACTCCTCTACCTACTCGACTACCCTAG | 3' Alexa Fluor 488 |
| **B13 Amplifier H1** | CCTGCTTTATGCTCAACATACAACCAGAAATGCGGCGTTGTATGTTGAGCATAAAGCAGGAAGGCGTTACCT | 5' Alexa Fluor 647 |
| **B13 Amplifier H2** | GCCGCATTTCTGGTTGTATGTTGAGCATAAAGCAGGAGGTAACGCCTTCCTGCTTTATGCTCAACATACAAC | 3' Alexa Fluor 647 |
| **B14 Amplifier H1** | GAGCGACCCTATATTTCTGCACAGAAGTTATACCGGCTGTGCAGAAATATAGGGTCGCTCGCTATTGACATT | 5' Alexa Fluor 488 |
| **B14 Amplifier H2** | CCGGTATAACTTCTGTGCAGAAATATAGGGTCGCTCAATGTCAATAGCGAGCGACCCTATATTTCTGCACAG | 3' Alexa Fluor 488 |
| **B15 Amplifier H1** | CCACAAGGTATCTCGAACACTCTCCAAATTGGCTACGAGAGTGTTCGAGATACCTTGTGGTGTGTTAATCTG | 5' Alexa Fluor 546 |
| **B15 Amplifier H2** | GTAGCCAATTTGGAGAGTGTTCGAGATACCTTGTGGCAGATTAACACACCACAAGGTATCTCGAACACTCTC | 3' Alexa Fluor 546 |
| **B17 Amplifier H1** | GTGGACACCTGCTAATCGGATGAGTGTTCGTTATCGCTCATCCGATTAGCAGGTGTCCACAACAAACAATCG | 5' Alexa Fluor 647 |
| **B17 Amplifier H2** | CGATAACGAACACTCATCCGATTAGCAGGTGTCCACCGATTGTTTGTTGTGGACACCTGCTAATCGGATGAG | 3' Alexa Fluor 647 |
| **qbc. 1 (for qPCR)** | TCTTGTGGAAAGGACGAAACACGTGATNNNNNNNNNNNNNNNGTCTGGAGCATGCGCTTTAG | 5' Amino C6 |
| **qbc. 2 (for qPCR)** | TACACGACGCTCTTCCGATCTCGTGATNNNNNNNNNNNNNNNTTGAAAAAGTGGCACCGAGT | 5' Amino C6 |
| **qbc. 3 (for qPCR)** | ACACGTCTGAACTCCAGTCACCGTGATNNNNNNNNNNNNNNNCGTATGCCGTCTTCTGCTTG | 5' Amino C6 |
| **qbc. 4 (for qPCR)** | GACAGTTCGAGTTTGAAGCGCGTGATNNNNNNNNNNNNNNNCTAGACGTGGGAGTGCATACT | 5' Amino C6 |
| **qbc. 5 (for qPCR)** | GAAAGATCTGGCTGCCATGCCGTGATNNNNNNNNNNNNNNNTCGCAAACCTGGTTGGAATCA | 5' Amino C6 |
| **qbc. 6 (for qPCR)** | AGATGACGTCGATTGTTGGTCGTGATNNNNNNNNNNNNNNNCATGGAGGTTGTGTCACCGTA | 5' Amino C6 |
| **qbc. 7 (for qPCR)** | TCAGGTGCATAGGAGTCAGCCGTGATNNNNNNNNNNNNNNNATGCTGTCAGTTCATGGCTCC | 5' Amino C6 |
| **sbc. 1 (for sequencing)** | TCTTGTGGAAAGGACGAAACA**CGTGAT**NNNNNNNNNNNNNNNGTCTGGAGCATGCGCTTTAG | 5' Amino C6 |
| **sbc. 2 (for sequencing)** | TCTTGTGGAAAGGACGAAACA**ATCACG**NNNNNNNNNNNNNNNGTCTGGAGCATGCGCTTTAG | 5' Amino C6 |
| **sbc. 3 (for sequencing)** | TCTTGTGGAAAGGACGAAACA**CGATGT**NNNNNNNNNNNNNNNGTCTGGAGCATGCGCTTTAG | 5' Amino C6 |
| **sbc. 4 (for sequencing)** | TCTTGTGGAAAGGACGAAACA**TTAGGC**NNNNNNNNNNNNNNNGTCTGGAGCATGCGCTTTAG | 5' Amino C6 |
| **sbc. 5 (for sequencing)** | TCTTGTGGAAAGGACGAAACA**TGACCA**NNNNNNNNNNNNNNNGTCTGGAGCATGCGCTTTAG | 5' Amino C6 |
| **sbc. 6 (for sequencing)** | TCTTGTGGAAAGGACGAAACA**ACAGTG**NNNNNNNNNNNNNNNGTCTGGAGCATGCGCTTTAG | 5' Amino C6 |
| **sbc. 7 (for sequencing)** | TCTTGTGGAAAGGACGAAACA**GCCAAT**NNNNNNNNNNNNNNNGTCTGGAGCATGCGCTTTAG | 5' Amino C6 |
| **Norm DNA (for sequencing)** | TCTTGTGGAAAGGACGAAACA**GCCAAT**NNNNNNNNNNNNNNNGTCTGGAGCATGCGCTTTAG |  |

The sequences of Ab bc/norm bc were underlined.
