# Supplementary material for: Modular DNA barcoding of nanobodies enables multiplexed in situ protein imaging and high-throughput biomolecule detection
Source: eLife. 2025 Jul 22;14:RP105225. doi: 10.7554/eLife.105225 (PMC12283080; doi:10.7554/eLife.105225)
Supplement: Supplementary file 4. [file elife-105225-supp4.docx]

**Supplementary File 4. Amino acid sequences of the constructs used in this study.**

| pET28a-His_6_-Ubiquitin-OaAEP1 (C247A):  MGSSHHHHHHSSGENLYFQGRPMQIFVKTLTGKTITLEVEPSDTIENVKAKIQDKEGIPPDQQRLIFAGKQLEDGRTLSDYNIQKESTLHLVLRLRGGARDGDYLHLPSEVSRFFRPQETNDDHGEDSVGTRWAVLIAGSKGYANYRHQAGVCHAYQILKRGGLKDENIVVFMYDDIAYNESNPRPGVIINSPHGSDVYAGVPKDYTGEEVNAKNFLAAILGNKSAITGGSGKVVDSGPNDHIFIYYTDHGAAGVIGMPSKPYLYADELNDALKKKHASGTYKSLVFYLEACESGSMFEGILPEDLNIYALTSTNTTESSWAYYCPAQENPPPPEYNVCLGDLFSVAWLEDSDVQNSWYETLNQQYHHVDKRISHASHATQYGNLKLGEEGLFVYMGSNPANDNYTSLDGNALTPSSIVVNQRDADLLHLWEKFRKAPEGSARKEEAQTQIFKAMSHRVHIDSSIKLIGKLLFGIEKCTEILNAVRPAGQPLVDDWACLRSLVGTFETHCGSLSEYGMRHTRTIANICNAGISEEQMAEAASQACASIP* |
| --- |
| pET21a-TP897-NGL-His_6_:  MQVQLVECGGGLVQAGDSLRLSCVASGRSLDGATMRWYRQAPGKEREFVAGIFWDEIGTEYADTAKGRFTISRDNAKNTIYLQMTNLRSEDTAMYYCNGLVFGGEYWGKGTLVTVSSNGLHHHHHH* |
| pET21a-TP1107-NGL-His_6_:  MQVQLVECGGGLVQPGGSLRLSCAASGFTFSDTWMNWVRQAPGKGLYWISAINPDGGNTAYADSVKGRFTISRDNAKNMVYLQMDNLRPEDTAMYYCAKGWVRLPDPDLVRGQGTQVTVSSNGLHHHHHH* |
| pET28a-His_6_-EGFP:  MGSSHHHHHHMVSKGEELFTGVVPILVELDGDVNGHKFSVSGEGEGDATYGKLTLKFICTTGKLPVPWPTLVTTLTYGVQCFSRYPDHMKQHDFFKSAMPEGYVQERTIFFKDDGNYKTRAEVKFEGDTLVNRIELKGIDFKEDGNILGHKLEYNYNSHNVYIMADKQKNGIKVNFKIRHNIEDGSVQLADHYQQNTPIGDGPVLLPDNHYLSTQSALSKDPNEKRDHMVLLEFVTAAGITLGMDELYK* |
| pET28a-His_6_-mCherry:  MGSSHHHHHHMVSKGEEDNMAIIKEFMRFKVHMEGSVNGHEFEIEGEGEGRPYEGTQTAKLKVTKGGPLPFAWDILSPQFMYGSKAYVKHPADIPDYLKLSFPEGFKWERVMNFEDGGVVTVTQDSSLQDGEFIYKVKLRGTNFPSDGPVMQKKTMGWEASSERMYPEDGALKGEIKQRLKLKDGGHYDAEVKTTYKAKKPVQLPGAYNVNIKLDITSHNEDYTIVEQYERAEGRHSTGGMDELYK* |
| *P_Tight_* (Tight TRE promoter)-EGFP-*PGK*-rtTA:  MVSKGEELFTGVVPILVELDGDVNGHKFSVSGEGEGDATYGKLTLKFICTTGKLPVPWPTLVTTLTYGVQCFSRYPDHMKQHDFFKSAMPEGYVQERTIFFKDDGNYKTRAEVKFEGDTLVNRIELKGIDFKEDGNILGHKLEYNYNSHNVYIMADKQKNGIKVNFKIRHNIEDGSVQLADHYQQNTPIGDGPVLLPDNHYLSTQSALSKDPNEKRDHMVLLEFVTAAGITLGMDELYK*  MSRLDKSKVINGALELLNGVGIEGLTTRKLAQKLGVEQPTLYWHVKNKRALLDALPIEMLDRHHTHFCPLEGESWQDFLRNNAKSFRCALLSHRDGAKVHLGTRPTEKQYETLENQLAFLCQQGFSLENALYALSAVGHFTLGCVLEEQEHQVAKEERETPTTDSMPPLLRQAIELFDRQGAEPAFLFGLELIICGLEKQLKCESGGPADALDDFDLDMLPADALDDFDLDMLPADALDDFDLDMLPG* |
| *3×CRE*-mCherry-PEST:  MVSKGEEDNMAIIKEFMRFKVHMEGSVNGHEFEIEGEGEGRPYEGTQTAKLKVTKGGPLPFAWDILSPQFMYGSKAYVKHPADIPDYLKLSFPEGFKWERVMNFEDGGVVTVTQDSSLQDGEFIYKVKLRGTNFPSDGPVMQKKTMGWEASSERMYPEDGALKGEIKQRLKLKDGGHYDAEVKTTYKAKKPVQLPGAYNVNIKLDITSHNEDYTIVEQYERAEGRHSTGGMDELYKLSHGFPPEVEEQAAGTLPMSCAQESGMDRHPAACASARINV* |
