# Supplementary material for: Modular DNA barcoding of nanobodies enables multiplexed in situ protein imaging and high-throughput biomolecule detection
Source: eLife. 2025 Jul 22;14:RP105225. doi: 10.7554/eLife.105225 (PMC12283080; doi:10.7554/eLife.105225)
Supplement: Supplementary file 5. [file elife-105225-supp5.docx]

**Supplementary File 5. Primers for qPCR-based BLISA.**

| **DNA barcode** | **primer** | **Sequence (5’ to 3’)** |
| --- | --- | --- |
| **qbc. 1 (for qPCR)** | Forward | TCTTGTGGAAAGGACGAAACACG |
|  | Reverse | CTAAAGCGCATGCTCCAGAC |
| **qbc. 2 (for qPCR)** | Forward | TACACGACGCTCTTCCGATC |
|  | Reverse | ACTCGGTGCCACTTTTTCAA |
| **qbc. 3 (for qPCR)** | Forward | ACACGTCTGAACTCCAGTCAC |
|  | Reverse | CAAGCAGAAGACGGCATACG |
| **qbc. 4 (for qPCR)** | Forward | GACAGTTCGAGTTTGAAGCGC |
|  | Reverse | AGTATGCACTCCCACGTCTAG |
| **qbc. 5 (for qPCR)** | Forward | GAAAGATCTGGCTGCCATGC |
|  | Reverse | TGATTCCAACCAGGTTTGCGA |
| **qbc. 6 (for qPCR)** | Forward | AGATGACGTCGATTGTTGGTCG |
|  | Reverse | TACGGTGACACAACCTCCATG |
| **qbc. 7 (for qPCR)** | Forward | TCAGGTGCATAGGAGTCAGC |
|  | Reverse | GGAGCCATGAACTGACAGCAT |
